# How Thick is the Air–Water Interface?—A Direct Experimental Measurement of the Decay Length of the Interfacial Structural Anisotropy

**DOI:** 10.1021/acs.langmuir.4c02571

**Published:** 2024-08-22

**Authors:** Alexander P. Fellows, Álvaro Díaz Duque, Vasileios Balos, Louis Lehmann, Roland R. Netz, Martin Wolf, Martin Thämer

**Affiliations:** † Fritz-Haber-Institut der Max-Planck-Gesellschaft, Faradayweg 4-6, 14195 Berlin, Germany; ‡ Instituto Madrileño de Estudios Avanzados en Nanociencia (IMDEA Nanociencia), 28049 Madrid, Spain; § Department of Physics, Freie Universität Berlin, Arnimallee 14, 14195 Berlin, Germany

## Abstract

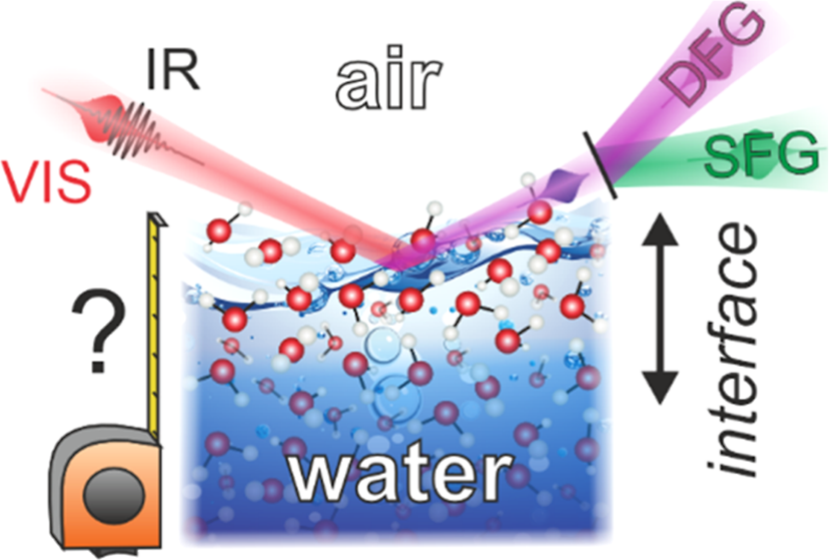

The air–water interface is a highly prevalent phase boundary impacting many natural and artificial processes. The significance of this interface arises from the unique properties of water molecules within the interfacial region, with a crucial parameter being the thickness of its structural anisotropy, or “healing depth”. This quantity has been extensively assessed by various simulations which have converged to a prediction of a remarkably short length of ∼6 Å. Despite the absence of any direct experimental measurement of this quantity, this predicted value has surprisingly become widely accepted as fact. Using an advancement in nonlinear vibrational spectroscopy, we provide the first measurement of this thickness and, indeed, find it to be ∼6–8 Å, finally confirming the prior predictions. Lastly, by combining the experimental results with depth-dependent second-order spectra calculated from ab initio parametrized molecular dynamics simulations, which are also in excellent agreement with this experimental result, we shed light on this surprisingly short correlation length of molecular orientations at the interface.

## Introduction

The air–water interface is ubiquitous in nature and serves as a useful model to study hydrophobic aqueous interfaces. Its importance is closely related to the unique characteristics of water in the interfacial region that is at the heart of numerous chemical processes in nature as well as industrial applications, with examples ranging from oceanic surfaces and atmospheric aerosols, to physiological membranes and electrochemical systems. This outstanding role of interfacial water has triggered an enormous number of experimental and theoretical investigations over the past several decades yielding exceptional insight into its structural properties.^1−7^ Nevertheless, some of the most fundamental aspects of the air–water interface still remain controversial or experimentally unverified, particularly the length-scale or “thickness” of the interfacial region.^8^

The presence of the phase boundary makes the interfacial region anisotropic with physicochemical properties that strongly deviate from the corresponding bulk values. This anisotropy consists of depth-dependent variations in molecular density, dielectric constant, as well as the distribution of molecular orientations and the number, strength, and dynamics of hydrogen bonds within the intermolecular network. The details of these variations and the length-scale of their decay govern the specific role water plays in the function and behavior of aqueous interfaces. For example, its dielectric properties influence its interactions with charges which play a role in chemical activity, ion transport, and electron transfer processes, while the density influences its viscosity and therefore many kinetic processes.^9−11^ Some of the most pertinent properties of water, however, are controlled by the specific orientational distributions of the water molecules and details of the hydrogen bond network i.e., its molecular and intermolecular structure. These include its solvation behavior and surface tension, which are critical in the thermodynamics underlying processes including uptake and transport mechanisms as well as chemical reactions.^12,13^ Evidently, knowledge of this depth-dependence to the structure and properties of interfacial water, and particularly the length over which they differ from the bulk, is central to understanding the functional behavior of interfaces which are widespread across many fields.

While the depth-dependent deviations in these different aforementioned properties are all obviously interconnected, they can in principle decay on different length-scales. In consequence, any value of the anisotropic thickness that is experimentally measured depends on the specific property being probed. For the air–water interface, the anisotropy decay in density and dielectric constant have been experimentally determined using techniques such as neutron reflectometry^14^ and ellipsometry,^15,16^ respectively. These studies indicate that their variation occurs over length-scales of ∼3–5 Å, and thus that the bulk density and dielectric constant are recovered very quickly. In contrast, a direct experimental measurement of the thickness of the anisotropic structure (molecular orientations and intermolecular connectivity) remains elusive.

The well-defined directionality and strength of hydrogen bonds in liquid water, along with its large molecular dipole, make the orientations of neighboring water molecules highly correlated. In pure bulk water the length-scale of these correlations is, however, somewhat contentious owing to the many influencing factors. On the one hand, these correlations are often considered to be contained within length-scales of ∼15 Å, thus with angular reorientation events of individual molecules triggering the surrounding molecules within ∼4–5 coordination shells to restructure.^17−20^ On the other hand, they have also been indicated to extend much further to 10s or even 100s of Å through acoustic coupling and long-range dipole–dipole and orientationally restrictive hydrogen bonding correlations.^20−23^ Such behavior is also highly debated upon the addition of charged electrolytes which both perturb the dipole orientations and distort the hydrogen bond network.^22,24−28^ In any case, with correlations being present over several coordination shells and potentially much further, it is clear that the hydrogen bond network can have a vast reach in generating long-range molecular order. It is nevertheless unclear whether the specific anisotropic molecular structure present at the interface causes orientational correlations similar to those in the bulk, or if they are much longer, or even shorter.

Current insight into this question primarily comes from molecular dynamics (MD) simulations which suggest a surprisingly short anisotropic structural thickness of ∼6 Å.^29−35^ This would mean that the bulk structure is already obtained by the fourth, or even third, molecular layer, and thus the reach of the hydrogen bond network of interfacial water on the molecular orientations below the interface is definitely shorter than in the bulk. This is especially interesting given that simulations have also indicated that the orientations of interfacial water molecules are actually highly correlated through expansive hydrogen bond connectivity, only that these correlations predominantly exist in-plane i.e., laterally within an overall isotropic 2D hydrogen bond network, and not normal to the interface.^36,37^ While this detailed structural view of the interface is very enlightening, it is also quite remarkable as it suggests that the interfacial molecules are somewhat detached from the bulk in an ultrathin layer. However, as it is known that results and interpretations from MD simulations can be highly sensitive to their choice of force field and specific parameter-sets, it is crucial to confront these simulations with independent experimental verification of this somewhat unexpected result for the anisotropic thickness.

An experimental technique that has been widely applied to aqueous interfaces is vibrational sum-frequency generation (SFG) spectroscopy.^13,38−53^ The strength of SFG for experimentally addressing this question is its sensitivity to molecular orientations that are encoded in the sign of the output signal phase, as well as the molecular environments and intermolecular interactions that control the specific line-shapes of the vibrational resonances.^54^ These can make it a selective probe of structural anisotropy as isotropic regions yield no signals under the electric dipole (ED) approximation owing to cancellation.^55,56^ SFG spectroscopy has been very successful in identifying specific interfacial water species such as water molecules with dangling bonds pointing into the air-phase (“free” OH).^38^ However, extracting information about the thickness of the structural anisotropy with SFG spectroscopy has proven to be a veritable challenge. Recently, Benderskii et al. used isotopic dilution SFG experiments to investigate the intramolecular coupling between the dangling OH and its associated hydrogen bonded mode to assess the hydrogen bond strength of the latter, ultimately showing it to be almost equivalent to that in the bulk.^8^ From this, they inferred that the structural anisotropy decays remarkably fast with depth. Later, Nagata et al. combined experimental measurements with simulations to investigate the anisotropy in the dielectric constant across the interface.^10^ Through simulations of the different contributions to the structural anisotropy, they show that the free OH stretch contribution and those from the hydrogen bonded modes must arise from locations within the interface with differing dielectric properties. From their results, they determine that the variation in dielectric constant across the interface predominately occurs over ∼1–3 Å. However, the length-scale of the structural anisotropy in this study was entirely derived from simulation and not extracted from the experimental results. Nevertheless, the observation of differing dielectric environments for the different structural motifs does suggest a short decay length of the structural anisotropy.

There are two major obstacles for accessing the thickness of the structural anisotropy using SFG spectroscopy: (i) the signals are integrated over depth and thus a single SFG measurement cannot directly yield information on this thickness, and (ii) when probing water, it is still unclear to what extent the ED approximation holds and the measured signals really originate exclusively from structurally anisotropic regions. Beyond the ED approximation, signals can also be generated through quadrupolar mechanisms, either due to the dielectric anisotropy at the interface (interfacial quadrupolar) or from the oscillating probing fields in the isotropic bulk (bulk quadrupolar). These signals could easily represent relevant contributions to the overall response, making the probe less selective to structural anisotropy. Whether such contributions from structurally isotropic regions are significant in the water response or not, has been a long-standing question in nonlinear spectroscopy and a clear answer has yet to be given.^57−60^ Addressing this question is obviously essential for obtaining precise information on the structural anisotropy decay.

In this contribution, we utilize our recently developed depth-resolved vibrational spectroscopy that overcomes these limitations.^61,62^ This technique allows us to perform depth-resolved analysis of the structural anisotropy of the air–water interface and provide a direct measurement of its thickness. This is made possible through the simultaneous phase-resolved measurement of two different second-order responses, namely sum- and difference-frequency generation (SFG and DFG), which allows for both the precise depth profiling of the signal sources on the sub-nm scale, as well as an unambiguous quantification of isotropic signal contributions. The experimental results are then compared to depth-resolved SFG spectra from ab initio parametrized MD simulations. Furthermore, we show that, through isotopic exchange measurements, the overall nonlinear response can be decomposed into a resonant and nonresonant contribution. From their independent analyses, we then unravel their different spatial origins and discuss the far-reaching impact of this finding on second-order spectroscopy measurements.

## Results and Discussion

In order to reveal the interfacial water structure and its evolution with depth, we use phase-resolved SFG–DFG spectroscopy across the ∼2300–2900 cm^–1^ frequency range to probe the O–D stretch vibration in D_2_O. By probing the resonant second-order response from the molecular vibrations, the different structural motifs within the interfacial region can be elucidated based on their characteristic line-shape features, and the depth information extracted from combining the SFG and DFG spectra. The general theory behind SFG spectroscopy can be found elsewhere in the literature,^54−56,63,64^ with the specifics underlying the depth-resolved SFG/DFG spectroscopy employed in this work detailed in previous publications.^61,62^ Here we only provide a brief discussion of its main features.

**Figure 1:**
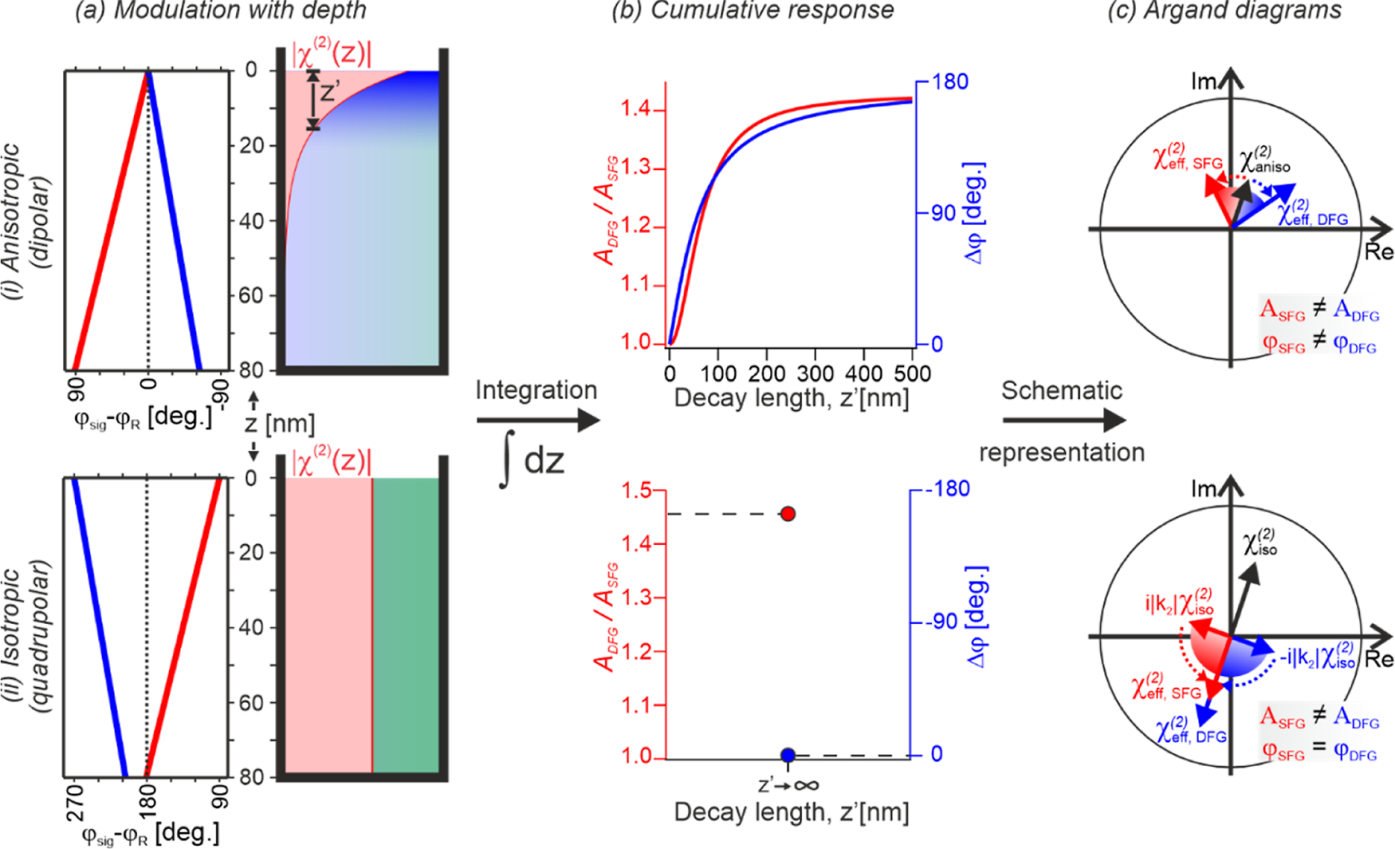
Schematic representation of the amplitude and phase modulations to the SFG and DFG responses for both (i) anisotropic (dipolar) and (ii) isotropic (quadrupolar) signals. (a) Graphical representations of the modulation of the phase and amplitude of the responses from chromophores as a function of depth. ϕ_sig_ represents the phase of the output signal from each depth and ϕ_*R*_ represents the resonant phase. (b) Graphs of the phase difference, Δϕ = ϕ_SFG_ – ϕ_DFG_, and amplitude ratio, *A*_DFG_/*A*_SFG_, of the cumulative (depth-integrated) responses as a function of the decay length of the signal contribution, *z*′. As the isotropic contribution does not decay [part (a) (ii), right panel], *z*′ → ∞ and only the limiting values are presented. (c) Schematic Argand diagrams of the effect of depth on the phase and amplitude of the effective (measured) SFG and DFG responses, χ_eff_^(2)^.

The generation of the SFG and DFG (s-polarized) signals is achieved by nonlinear frequency mixing between two laser pulses, namely a mid-infrared (p-polarized) pulse that probes vibrational resonances and a visible upconversion (s-polarized) pulse with photon energy far from any sample resonance. The phases of these sample responses are determined interferometrically using SFG and DFG reference pulses (local oscillators, LO) that are linearly reflected by the sample surface. This plane of linear reflection (PLR) serves as reference position (*z* = 0) for our depth-resolved studies. Any second-order electric dipolar signal that arises from structural anisotropy and originates exactly from this depth plane, generates SFG and DFG spectra that precisely coincide in both phase and amplitude. However, signals from any deeper layers contributing to the dipolar response (*z* > 0) are phase-shifted in opposite directions for SFG and DFG (see Supporting Information or refs (61 and^62^) for details), as shown in Figure 1a(i) for selected pathways. This phase-shift arises from the added propagation of the input and output beams and increases linearly with depth. The modulated phases lead to a phase difference between the integrated SFG and DFG responses that approaches 180° for large decay lengths, *z*′, as shown in Figure 1b(i). The apparent phase difference between SFG and DFG is hence a direct measure of the decay length of the structural anisotropy. An example of the distinct phase difference introduced between SFG and DFG from nonzero depth is given in a previous study on a model system.^62^ In addition, a further demonstration of the technique is given here in the Supporting Information for the case of charged aqueous interfaces which have signal contributions from extended depths due to field-induced water reorientation.

In contrast to the anisotropic (dipolar, but also interfacial quadrupolar) response, any contribution arising from isotropic bulk environments (bulk quadrupolar) has distinctly different characteristics. As shown in Figure 1a(ii) and the Supporting Information, this intrinsic response is inherently shifted by +90° for SFG and −90° for DFG. The corresponding integration over depth further phase-shifts the SFG and DFG responses, leading to a decreasing phase difference that tends to 0°. As the isotropic signals always originate from the entire bulk, the phase difference between their integrated SFG and DFG responses must be precisely zero, unlike the dipolar case, as indicated in Figure 1b(ii). In addition to the phase-shift, any integration also leads to differing amplitudes for SFG and DFG, which only become significant for depth values ≫10 nm (see Supporting Information). Such a situation is obviously given for any isotropic contribution [as *z*′ → ∞, see Figure 1b(ii)] but typically not the case for the anisotropic response when considering its expected nanoscale decay length. Generally, the anisotropic contribution can, in principle, yield different phases and amplitudes for SFG and DFG, however, only the phase difference is typically significant. On the other hand, the isotropic contribution presents no phase difference but an amplitude ratio clearly deviating from unity [as depicted in the schematic phase diagram in Figure 1c(ii)].

**Figure 2:**
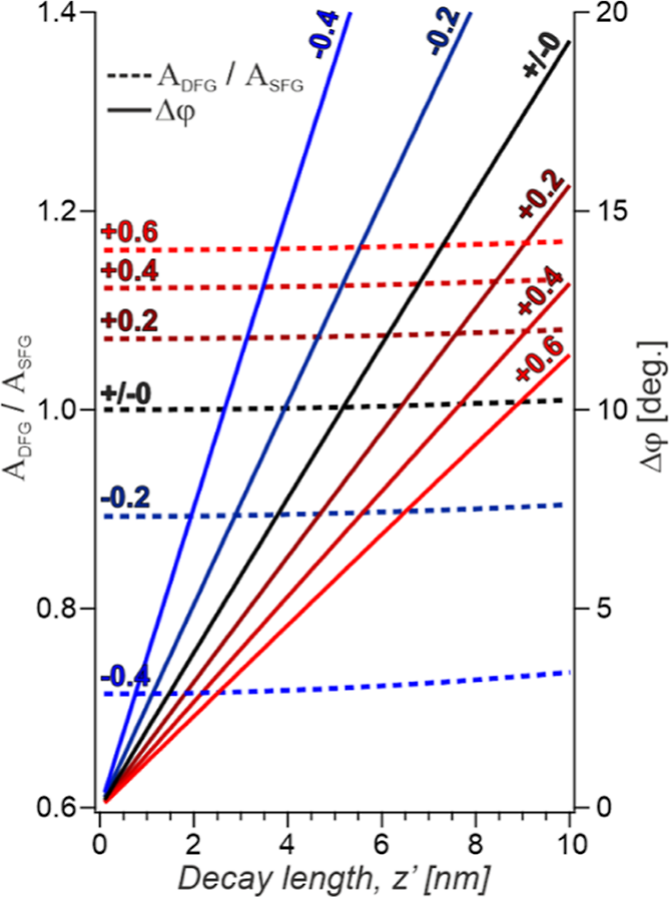
Plot of the amplitude ratio and phase difference of the combined response comprising an anisotropic contribution and varying relative isotropic contributions. The ratio of the amplitudes of the isotropic to anisotropic contributions are indicated on each trace.

Based on these differing characteristics, anisotropic and isotropic signal sources can be separated, and the purely anisotropic decay can be determined. Particularly straightforward is this determination for the typical cases where the anisotropy decay is rather small (<10 nm) as depicted in the graphical representation shown in Figure 2. Here, the theoretical overall amplitude ratio and phase difference are shown as a function of the anisotropic decay length considering different relative isotropic contributions to the combined response. From this, it becomes clear that the deviation of the amplitude ratio from unity exclusively speaks to the relative proportion of isotropic component, while the phase difference is modulated by both aniso- and isotropic responses. Therefore, the exact decay length of the structural anisotropy can be extracted by first determining the isotropic contribution based on the amplitude ratio and using this to correctly transform the measured phase difference into the corresponding value of *z*′.

**Figure 3:**
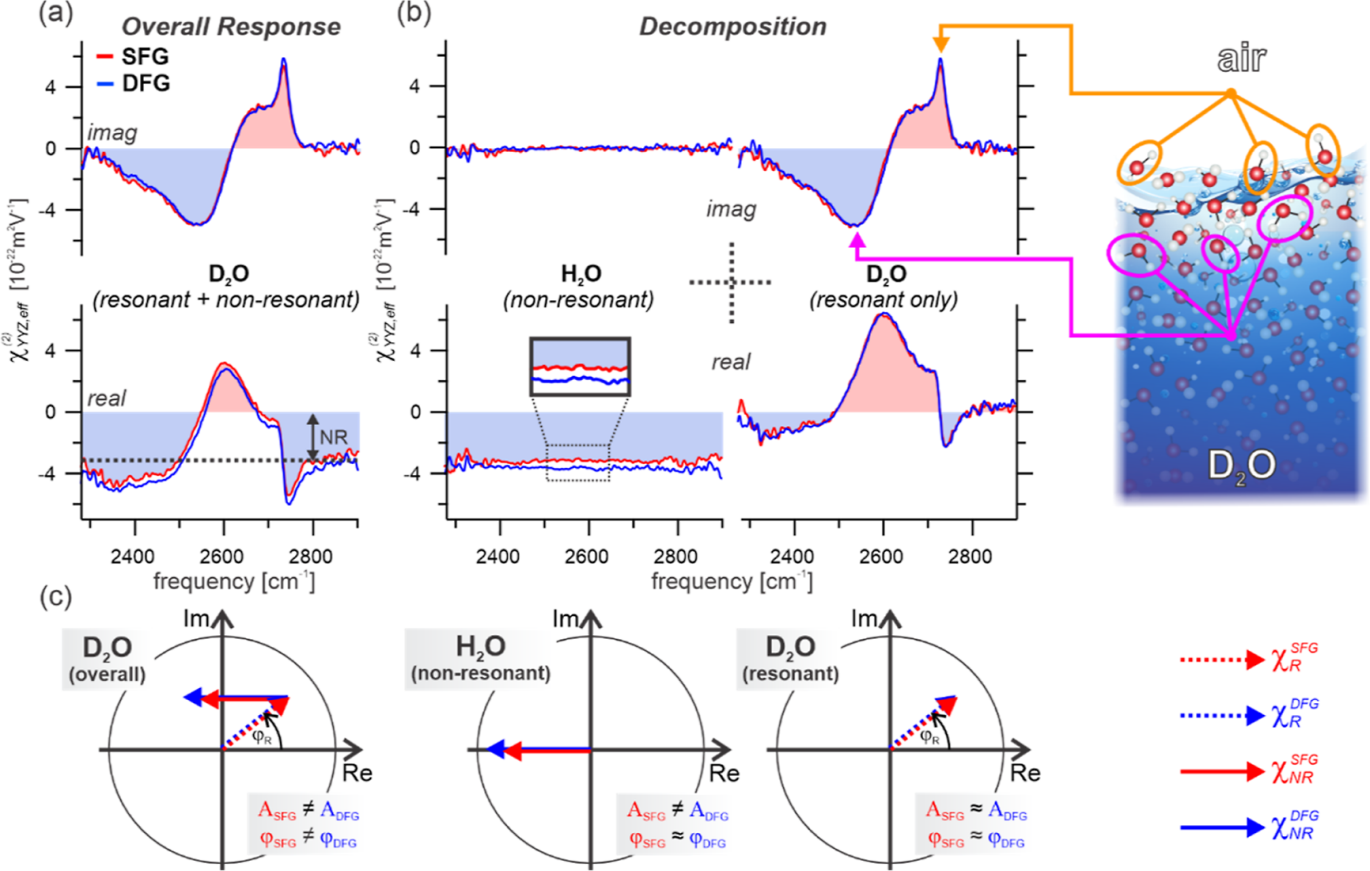
SFG and DFG spectra of the air–water interface in the SSP polarization combination. (a) real and imaginary parts of the second-order response from D_2_O given in absolute units. The dashed line in the real part indicates the nonresonant contribution to the spectra (NR). (b) real and imaginary parts of the purely nonresonant H_2_O response as well as the D_2_O response having subtracted that from H_2_O, thus only representing the resonant contribution. The spatial origin of structural motifs giving rise to the two most significant stretching bands, namely the “free” OD at 2740 cm^–1^ and H-bonded OD at ∼2540 cm^–1^, are schematically indicated. (c) Schematic Argand diagrams of the three spectra presented in (a) and (b), emphasizing any differences between the amplitudes and phases of the SFG and DFG responses.

With this analytical procedure in hand, we turn to the experimental results from the D_2_O-air interface shown in Figure 3a. The obtained SFG and DFG spectra are split into their real and imaginary parts, corresponding to the dispersive and absorptive line-shapes, respectively. Before performing the depth analysis, we briefly discuss the obtained resonant line-shape. The spectra exhibit four clearly distinguishable absorption bands, two negative contributions at ∼2400 and 2540 cm^–1^ which highly overlap, along with two positive bands, one being a sharp feature at 2740 cm^–1^, and a broader shoulder to this band at ∼2680 cm^–1^. The specific resonant frequencies of the stretching modes are particularly sensitive to hydrogen bond strength, with them becoming increasingly red-shifted for stronger intermolecular bonds.^65^ Therefore, each structural motif in liquid water possesses a characteristic vibrational response which enables their identification. The positive sharp band at 2740 cm^–1^ is assigned to the free OD stretch where the positive sign of the resonant peak indicates a preferential “pointing up” orientation of this water species, in accordance with its interpretation.^65−68^ By contrast, the two overlapping negative bands between 2300 and 2600 cm^–1^ originate from hydrogen bonded O–D stretch vibrations having transition dipoles pointing down on average.^65−68^ Furthermore, the assignment of the positive shoulder at 2680 cm^–1^ overlapping with the free OD has been highly contentious over the past several years, although the positive sign indicates it has a general direction toward the air-phase. Its origin has been suggested as the antisymmetric OD stretch arising from intramolecular coupling from D_2_O molecules presenting one acceptor and two donor hydrogen bonds,^8,46,69^ but more recently has been assigned to a Fermi resonance of the free OD with a combination band mixing the hydrogen-bonded OD stretch with a low-frequency intermolecular vibration.^70^ Finally, in addition to the resonant line-shape, it is important to note the presence of a significant nonresonant contribution arising from electronic interactions which can clearly be seen by the large negative offsets in the real parts of the spectra.

Upon comparison of the SFG and DFG spectra, they show remarkable similarities, but are clearly not identical. As signals purely arising from the immediate phase boundary (zero depth) must yield exactly equal SFG and DFG spectra, this discrepancy suggests that the signals must have contributions from deeper down, further from the interface (nonzero depth). Furthermore, as the discrepancies are quite clearly observed by eye, this suggests that there are signals from depths that are significant compared to the coherence length (here ∼50 nm). On closer inspection, the vast majority of the difference is present in the real parts (Figure 3a, lower panel) which appear to only differ by a constant offset, that is the real part of the DFG response seems to have a larger (negative) offset from zero. Interestingly, this suggests that the difference arises solely from the nonresonant contribution as it seems entirely independent of frequency and predominantly arises in the real part. Apparently, the resonant and nonresonant contributions seem to report on different depth profiles, necessitating their separation. This is achieved through isotopic exchange experiments by measuring the analogous spectra for H_2_O. Since both isotopologues have identical electronic structures, it is reasonable to treat their nonresonant contributions, which are dominated by electronic interactions, as being equal. The H_2_O spectra are depicted in Figure 3b, showing that they indeed precisely reproduce the same apparent negative offset as in D_2_O. Based on this, the overall spectra can be fully decomposed into their pure resonant and nonresonant contributions, as shown in Figure 3b.

With the resonant and nonresonant contributions separated, we see that the purely resonant SFG and DFG spectra (Figure 3b, right-side) almost perfectly overlap, and thus that this contribution reports on a short anisotropic decay (small phase difference) and has no significant isotropic contribution (equal amplitudes). The purely nonresonant spectra also show little phase difference (Figure 3b, left-side, imaginary parts are both ∼0), but, in contrast, clearly feature a deviation in their amplitudes (Figure 3b, left-side, offset in real part). Following Figure 2, this demonstrates that the nonresonant contribution must possess a considerable isotropic component (from the bulk). For its quantification within both signal contributions, we assess the amplitude ratios from each. For the nonresonant contribution, this can be done with high precision as it is independent of frequency and thus can be spectrally fitted with a constant value. The obtained ratio of 1.148 ± 0.002 reveals that ∼34% of the SFG (and ∼42% of the DFG) nonresonant response originates from the isotropic bulk, representing a remarkably large bulk contribution (see Supporting Information for details). In contrast, the average value of the amplitude ratio for the resonant contribution is 1.00 ± 0.04. This mean value of precisely 1 indicates that there is no considerable isotropic contribution, consistent with the observation of highly overlapping spectra. However, the larger standard deviation compared to the nonresonant contribution reports a larger uncertainty for this assessment. Nevertheless, the size of the standard deviation in the average value can be used to put an upper bound on a possible isotropic contribution, showing that it must be <10% of the overall resonant response. Therefore, the resonant contribution is clearly dominated by the anisotropic dipolar signal. The observed differences between the SFG and DFG resonant and nonresonant contributions are well-represented by the schematic Argand diagrams in Figure 3c.

**Figure 4:**
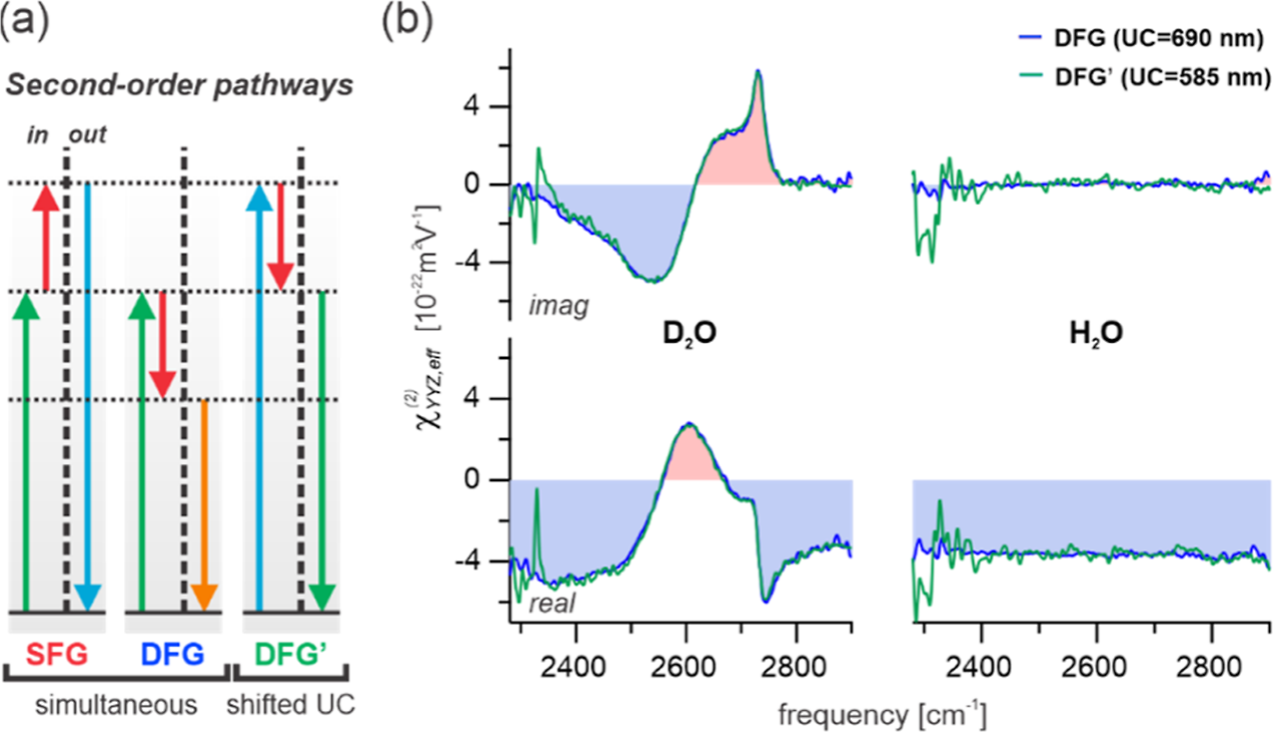
Dispersion test for the observed amplitude ratio in the nonresonant response. (a) Schematic energy level diagrams of the SFG and DFG pathways produced simultaneously as well as for a separate DFG pathway, DFG′, using a shifted upconversion frequency at the initial SFG frequency. (b) Comparison of the resulting DFG and DFG′ spectra for both D_2_O and H_2_O.

Evidently the determination of the isotropic contributions, and thus the anisotropic depths, is highly sensitive to the accuracy at which the amplitude ratio is determined. Therefore, any possible other sources of deviations, such as dispersion effects must be ruled out. The energy level diagram in Figure 4a shows that SFG and DFG involve different frequencies, and thus dispersion could potentially by present, although is typically insignificant.^61^ Nevertheless, we test experimentally for any impact from dispersion by measuring a separate DFG response (labeled DFG′, see Figure 4a) using a shifted upconversion at the original SFG frequency to compare to the original DFG response, as shown in Figure 4b. If dispersion is zero, the amplitude ratio between the two nonresonant responses should be close to unity, with a slight deviation arising from a small modulation of the apparent isotropic contribution (see Supporting Information). The theoretically derived ratio for this case is 0.988. The presence of dispersion effects should meanwhile appear as a clear deviation from this value. From a comparison of the fitted values from the experimental results, we obtain a measured amplitude ratio of 0.991, which is in remarkable agreement with the predicted value. A summary of this comparison is given in Table 1. These values clearly show that dispersion effects are indeed negligible in these experiments.

**Table 1:** Comparison between Measured and Predicted Values of the Ratio of H_2_O Non-Resonant Amplitudes for DFG Responses Measured with Upconversion Beams at 585 and 690 nm^a^

susceptibility ratio	measured	predicted
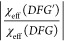	0.991 ± 0.003	0.988 ± 0.010

a The uncertainty in the measured value arises from the fit of the non-resonant response whilst that for the predicted value sources from the uncertainty in the relative isotropic contribution, and thus from the measured amplitude ratio between SFG and DFG.

**Figure 5:**
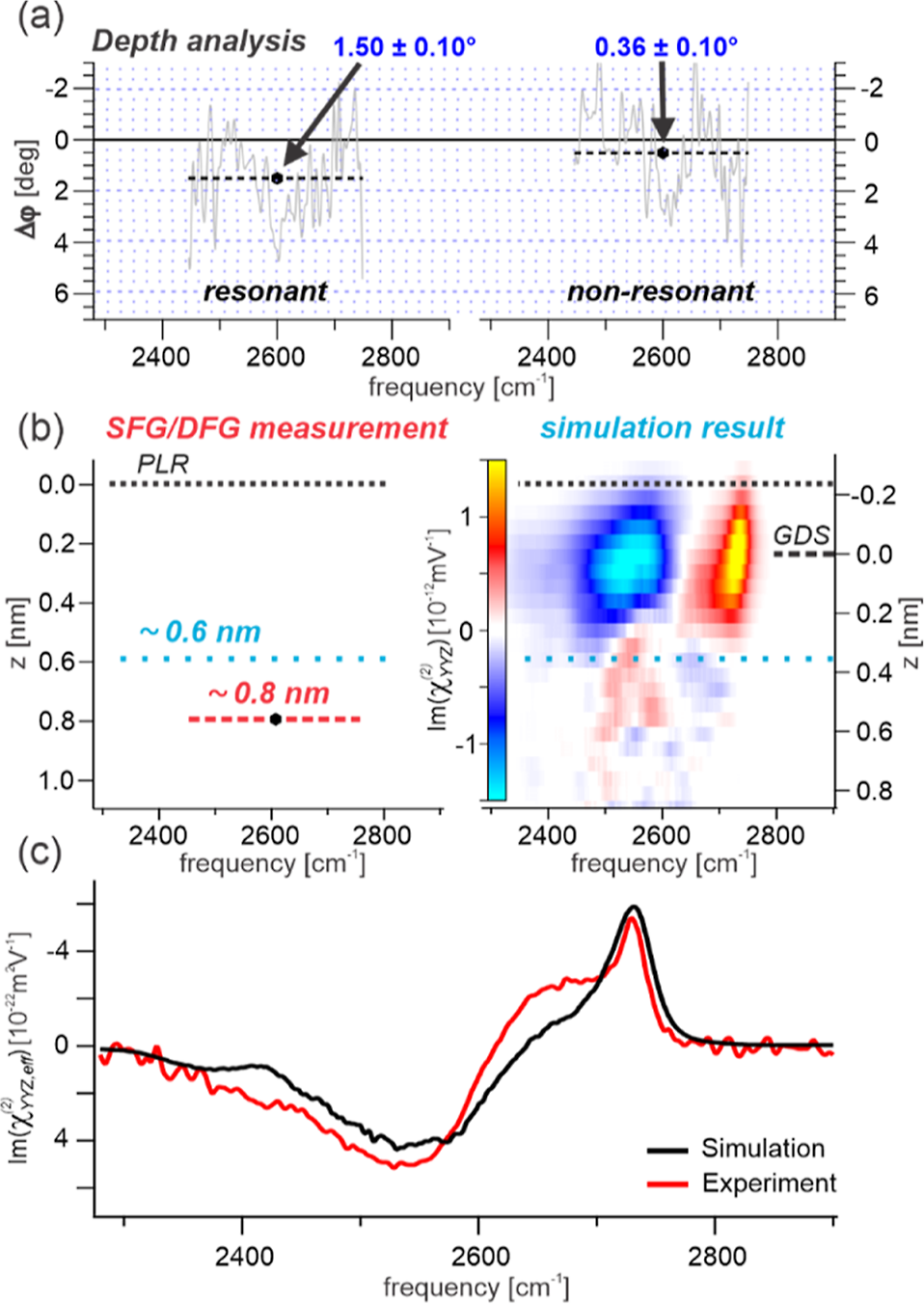
Depth analysis of the resonant and nonresonant responses. (a) Plotted phase difference between SFG and DFG for each contribution. The raw data in each plot have been fitted with a constant to extract a specific depth value. (b) Comparison between the experimentally obtained depth value and that from depth-dependent SFG spectra calculated from ab initio parametrized MD simulations. (c) Integrated SFG response overlapping with the experimentally obtained purely resonant SFG D_2_O spectrum, with both shown in absolute units. (PLR: plane of linear reflection, GDS: Gibbs dividing surface. Note the different zero positions, see Supporting Information for details.).

Based on these findings, we can now extract the decay length of the structural anisotropy from the phase difference between SFG and DFG depicted in Figure 5a. The resonant phase difference spectrum is very close to zero, but generally slightly positive, which corresponds to locations just below the interface. The average values of the phase differences across all frequencies within the bandwidth of the resonances are 1.50 ± 0.10° for the resonant contribution and 0.36 ± 0.10° for the nonresonant contribution. With their respective proportions of isotropic contributions given above, these phase differences can be converted into their corresponding decay lengths of the anisotropic contributions, yielding values of 7.7 ± 1.0 Å for the resonant component and 3.1 ± 0.9 Å for the nonresonant component. It is important to note that the stated uncertainties in these values are derived from the combination of the uncertainty in the fit for the phase difference and the uncertainty in the conversion factor between phase difference and decay length which stems from the measured size of the isotropic contribution. They are hence neglecting any systematic errors as well as any inherent frequency dependence to the phase difference, which could be present in the resonant contribution. Therefore, in reality, the confidence intervals of the decay lengths for each contribution are likely broader and could well span several Ångströms. Despite this uncertainty, with both values being clearly below 1 nm, it can be safely concluded that no significant structural anisotropy extends beyond the first ∼3 molecular layers.

The experimentally obtained mean value for the decay length of the resonant response (7.7 Å) can be directly compared to the predictions from MD simulations (which only include the vibrationally resonant contributions). The right panel of Figure 5b shows the depth-dependent second-order susceptibility in absolute units extracted from MD simulations. A quantitative comparison of the overall integrated response with the experimentally obtained purely resonant spectrum is then presented in Figure 5c. The simulation results clearly show the same resonant features as in the experiment, namely the two overlapping negative (blue) low-frequency modes as well as the positive (red) free OD stretch at 2740 cm^–1^ and its low-frequency shoulder, along with excellent agreement in absolute amplitudes. Figure 5b then reveals that both positive and negative spectral features source from essentially the same interfacial region, with equal onsets and termination depths. On closer inspection within this region, there is a clear redshift with increasing depth for both spectral features, reflecting the gradient in hydrogen bond strength. Overall, the simulated resonant features are contained within a thickness of 6 Å (from *z* = −2 Å down to 4 Å). A direct comparison to the experimentally obtained value (∼7.7 Å) places the experimental and simulation results within 2 Å of each other i.e., within a single molecular layer, and thus in consensus within the uncertainty of each method. The numerically predicted *z*-dependent susceptibility in Figure 5b agrees qualitatively with previous numerical approaches based on the variation of a depth-dependent threshold function^33,35,57^ or normal modes.^29^ Such a threshold function has also been used to obtain depth-resolved SFG signals at lipid–water interfaces and in the presence of external electric fields.^71,72^ All of these works, however, investigate H_2_O and not D_2_O.

As shown above, the resonant and nonresonant contributions report slightly different mean values for the anisotropy decay (7.7 ± 1.0 versus 3.1 ± 0.9 Å). This raises the question of which of the values more accurately describes the structural anisotropy. The resonant response is dominated by vibrational interactions and hence should be highly sensitive to both the distribution of molecular orientations and any anisotropy in the hydrogen bond connectivity. These sensitivities are clear as the resonant response probes the orientation-dependent anisotropy in the vibrational potential (i.e., Morse potential). Therefore, subtle changes in the orientational distribution can lead to large changes in the amount of signal cancellation, and thus also in the magnitude of the overall response. Furthermore, changes in hydrogen bonding significantly perturb the vibrational potential, resulting in large frequency shifts.^73^ This makes the overall response also sensitive to the anisotropy in the intermolecular environments and, for example, can further alter the amount of signal cancellation from oppositely oriented molecules (if they have different hydrogen bonding environments). Furthermore, in the case of the O–D stretch, the resonant response contains no significant isotropic quadrupolar contributions (as shown earlier from the SFG/DFG amplitude ratio). Clearly, therefore, any anisotropy of the molecular structure within the extensive hydrogen bond network is expected to be the predominant factor influencing the length-scale obtained from the resonant response. On the other hand, the nonresonant response is dominated by electronic interactions and is thus generally related to the asymmetry of the electron cloud^74^ and could well be less sensitive to molecular orientations and any intermolecular interactions, and thus show a different evolution with depth. Here, it is shown that the water nonresonant response even contains substantial isotropic contributions which compose almost half of the overall signal. Thus, a significant portion of the signal is clearly not reporting on any anisotropic aspects of the interface. Furthermore, it is even possible that the overall nonresonant response is highly insensitive to structural anisotropy altogether. Given its substantial isotropic contribution from bulk quadrupolar sources, is it not unreasonable to expect that anisotropic (interfacial) quadrupolar sources may also be significant contributors since they originate from the same fundamental mechanism.^58^ Unlike the dipolar mechanism which solely reports on the structural anisotropy, the anisotropic quadrupolar contributions arise from the discontinuity of fields at the interface and thus primarily report on the length-scale of the dielectric anisotropy.^55^ In this context, the value of ∼3.1 Å obtained here agrees remarkably well with previous measurements of the thickness of the dielectric variation across the interface.^9,10^ It is thus entirely possible, that the dipolar contribution only represents a minor contributor to the overall nonresonant response of water (see Supporting Information for a more detailed discussion).

Based on the discussion above, we conclude that the resonant response is indeed a far better probe of the structural anisotropy. Furthermore, as the extracted length-scale is in good agreement with the simulation result, both experiment and theory report a complementary view of the structural evolution of water at the interface with air. Hence, it can be conclusively stated that the effect of the phase boundary on the out-of-plane molecular structure does diminish remarkably quickly (∼6–8 Å) and seems to concern only the first 3 molecular layers. This means that the scale of the anisotropic structure (both orientational correlations and hydrogen bond connectivity normal to the interface) of interfacial water is rather shorter than the length scale of the correlations in the isotropic bulk. While this result follows theoretical predictions well,^29−37^ it is still somewhat surprising and raises questions about the underlying factors that dominate the structural anisotropy at the interface. First, the hydrogen bond connectivity is clearly reduced/weakened in the first molecular layer at the interface, making it unfavorable in terms of its free energy (as evidenced by the considerable surface tension of water) and thus there is a significant driving force toward retaining the bulk connectivity as quickly as possible. From this perspective, the observed fast decay is in line with expectations. This driving force, however, does not necessarily impose a loss in any preferential orientation. Of course, the lower connectivity of the interfacial molecules suggests that they inflict less orientational restriction on subsequent layers, but such an effect only considers the impact of individual molecules, and not their cumulative alignment and the resulting electrostatic effects from oriented dipoles. If the interface induces a substantial out-of-plane preferential dipolar direction, even if this oscillates between consecutive molecular layers, the alignment of dipoles could be expected to impose similar preferential orientations in the layers beneath, and thus exhibit longer range correlations. On the other hand, the lower hydrogen bonding connectivity at the interface enables greater orientational freedom, and thus a gain in entropy, in agreement with the well-known effect of decreasing surface tension on increasing temperature. Such an entropic gain can, however, only be realized through short orientational correlations. Therefore, since larger correlations are not observed, this suggests that the entropic gain dominates over the electrostatic correlation effects, and thus that the hydrogen bond network is the predominant factor influencing the structural anisotropy at the interface.

Beyond these insights into the water structure, our findings also have far-reaching consequences for nonlinear optical measurements on aqueous interfaces. As shown above, the nonresonant response of water is both clearly not a selective probe for anisotropic environments and may well be even fairly insensitive to structural anisotropy. Since a central pillar of second-order measurements is the anisotropic selectivity, these findings place substantial constraints on the interpretation of second-order spectra from aqueous interfaces. This concerns, in particular, intensity SFG approaches where the resonant contribution cannot be isolated via simple subtraction but require typically contentious multiparameter fits, and second harmonic generation spectroscopy that entirely relies on the interpretation of nonresonant signals. In contrast, the resonant response has been demonstrated to be a good marker for structural anisotropy, and has been shown to be extremely localized to the surface. This makes both amplitude and phase of the resonant response highly insensitive to the specific experimental setting, with any effects from incident beam angle or Fresnel factors being separable from the measured response. The reported resonant spectrum can thus be considered as an intrinsic property of the water surface and could be a useful reference for comparisons between different experimental set-ups as well as for the result of simulations. On the other hand, the nonresonant response will change with experimental settings due the combination of isotropic and anisotropic contributions (as discussed in Supporting Information). It is therefore not an intrinsic property of the system. However, this dependency is only manifested in its amplitude, with its phase being highly insensitive to the specific settings. This is due to its isotropic contribution being entirely real and the anisotropic component reporting a very small depth. This result is especially important as the potential role of the nonresonant water response as a phase reference in second-order measurements has been controversial, with no agreement on its true phase.^75−77^ Nevertheless, our results conclusively show that the phase of the nonresonant response is very close to ±180° (specifically, −179.6° for SFG) and is almost entirely insensitive to the specific experimental settings. This contrasts strongly to quartz which is by far the most commonly used phase and amplitude reference, but has been shown to have a significant phase deviation from the typically assumed phase of ±90°.^78,79^ On the other hand, unlike quartz, the nonresonant water response is clearly not a good amplitude reference. As such, the combination of water and quartz would make an excellent reference pairing for phase and amplitude measurements, respectively.

## Conclusions

In conclusion, we report the experimentally determined thickness of the structural anisotropy of the air–water interface using a newly developed second-order vibrational spectroscopy, finding it to be ∼6–8 Å. The obtained decay length is compared to depth-resolved SFG spectra calculated from ab initio parametrized MD simulations, showing excellent agreement. These combined results report on a remarkable short length-scale for both the correlation of molecular orientations normal to the interface and recovery of bulk hydrogen bond connectivity, covering only 3 molecular layers. This ultrashort correlation length-scale highlights the important role of the interfacial entropy alongside the loss in hydrogen bonding connectivity in dictating the molecular structure at the interface. Furthermore, we show that the resonant signal from the OD stretch vibration is indeed a selective probe of the structural anisotropy whereas the nonresonant (electronic) contribution is found to be little selective as it contains significant contributions from the isotropic bulk. This result underlines the importance of a careful analysis of the mechanistic origin of the signals in second-order spectroscopy and raises fundamental questions about the correct interpretation of results from nonresonant studies of aqueous interfaces. This also includes resonant SFG studies if the nonresonant contribution is not properly accounted for. Nevertheless, we have demonstrated that the presented depth-resolved vibrational spectroscopic technique allows these challenges to be overcome and obtain precise insight into the evolution of the structural anisotropy with depth in such systems.

## Materials and Methods

### Sample Preparation

The spectroscopic measurements of the air–water interface were performed on both H_2_O (Milli-Q, 18.2 MΩ·cm, <3 ppb TOC) and D_2_O (VWR Chemicals, 99.9% D). The water was contained in a custom-made Teflon (PTFE) trough which was cleaned overnight with Piranha solution (3:1 sulfuric acid to 30% hydrogen peroxide solution) and thoroughly rinsed with ultrapure water prior to use. Warning: Piranha solution is highly corrosive and an extremely powerful oxidizer. Great care must be taken with its preparation and use.

### Spectral Acquisition

The SFG and DFG spectra were recorded in the time-domain using a home-built nonlinear interferometer, the details of which can be found elsewhere.^80^ In short, two 1 kHz 800 nm outputs (4 and 3 W) of a Ti:sapphire laser (Astrella, Coherent) are used to pump two optical parametric amplifiers (TOPAS, Light Conversion). In the first, the pump beam is split into signal and idler, with the signal output being taken and frequency-doubled using a BBO crystal to use as the upconversion beam. The second TOPAS is used to generate tunable mid-IR via a DFG unit. Part of the IR beam (∼5%) is taken off using a beamsplitter and combined collinearly with the upconversion, to generate LO beams from z-cut quartz. The fully collinear output containing the upconversion and both SFG and DFG reference beams (LOs) are then recombined collinearly with the remaining ∼95% of the IR after an interferometric translation stage to control the relative timing of the pulses. The combined beam is then sent toward the sample via an oscillating mirror to split the beam in two and perform shot-to-shot referencing using a reference z-cut quartz crystal. Both beam paths are focused toward the samples at a 70° incidence angle from the surface normal after which they are recombined, spectrally filtered and detected using silicon photodiodes implementing balanced detection.

The spectra were measured with fast-scanning over the time delays of −500 to 6000 fs to ensure sufficient frequency resolution in the resulting spectra. The presented spectra using the 690 nm upconversion represent the average across 30,000 measurements taken from 3 different samples and those using the 585 nm upconversion represent the average from 20,000 measurements across two different samples. The spectra from each sample were compared and showed excellent reproducibility. Spectra were recorded in the ∼2300–2900 cm^–1^ frequency range, thus covering the O–D stretching region. This region was selected instead of the O–H stretching region purely for experimental reasons. First, the IR generation from the TOPAS is significantly more efficient in the O–D region, thus giving access to greater IR powers. Second, the suppression of any parasitic contributions from the collinear beam geometry requires less optical material in the lower frequency range, and is thus easier to implement and ensure good quality spectra. Finally, due to the effective mass difference, the O–D stretches cover a narrower frequency range than the O–H stretches. Therefore, covering the entire region within the envelope of the IR is more straightforward and yields better signal-to-noise across the entire resonant line-shape, especially given that the IR bandwidth generated from the TOPAS is larger at lower frequencies.

During measurement, the entire optical path up to and including the sample is purged with dry, CO_2_-free air to minimize any atmospheric absorption. Additionally, to ensure no change in the beam path or relative position of the sample surface, the height of the sample is continuously corrected for evaporation using an automated z-stage. Given that the measurement of the air–water interface necessitates a pure, clean surface, it is imperative that no surface contamination affects the results. However, even if some contamination to the surface occurs during the measurements, the local heating from the IR beam in the vicinity of the laser spot causes any surface-active species to migrate away via a substantial Bénard–Marangoni force.^81^ Therefore, only at relatively high surface concentrations would any contaminants enter the probed surface region and thus alter the obtained spectra.

### Amplitude and Phase Correction

The acquired SFG and DFG spectra were referenced in amplitude using the spectra from a *z*-cut quartz sample to remove the effect of the IR envelope. They were then further corrected for Fresnel factors and the beam geometry and converted into absolute units using the known susceptibility of quartz (0.6 pm V^–1^).^63^ This quartz measurement also gives an absolute phase reference for the sample spectra, which was taken to be ±90°, assuming the signal from quartz is an entirely nonresonant bulk dipolar response starting from the surface. As discussed in the main text, however, although this amplitude correction is valid, the assumed phase from quartz is slightly inaccurate.^79^ For this reason the phases were corrected using a further SFG/DFG measurement of an octadecyltrichlorosilane monolayer self-assembled on fused silica. Given that the signals arise from the terminal methyl groups in such a sample, they effectively have no depth and thus the phase of their SFG and DFG response should be precisely equal. A more detailed discussion of this phase correction is given below when comparing zero positions for the depth.

### Calculation of Spatially Resolved SFG Spectra from Molecular Dynamics Simulations

The theoretical prediction of SFG- and DFG spectra is based on the second-order polarization given in eq 1(55,56,82)
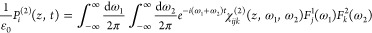
1where χ_*ijk*_^(2)^ (*z*,ω_1_,ω_2_) is the second-order response function and *F*_*j*_^1^(ω_1_) and *F*_*k*_^2^(ω_2_) are external electric fields which represent D or E fields.^83,84^ We employ the Einstein sum convention and *i,j,k* ∈ {*x,y,z*} are Cartesian indices. As the system is homogeneous in the *xy*-plane, *z*-polarized external fields correspond to electric displacement fields ε_0_^–1^*D*_*z*_ (*t*) and *x*- or y-polarized fields correspond to electric fields *E*_*x*/*y*_ (*t*). As we are interested in the nonlinear response of the system to monochromatic fields, we use eq 2
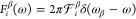
2where  is the vectorial amplitude of the external field and the index β ∈ {IR, VIS} distinguishes the IR from the visible (VIS) field source. As the VIS field does not resonate with the system, the dependence of the nonlinear response function on ω_VIS_ can be neglected, i.e., χ_*ijk*_^(2)^ (*z*, ω_VIS_, ω_IR_) ≈ χ_*ijk*_^(2)^ (*z*, ω_IR_). Consequently, we can write the nonlinear response of the system as in eq 3.
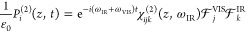
3

In the SSP polarization combination, the corresponding position and frequency-dependent susceptibility for SFG and DFG is defined by eq 4
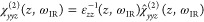
4where ε_*zz*_^–1^ (*z*, ω_IR_) is the inverse dielectric profile, which can be extracted by methods described earlier.^85^ The difference between the response function χ_*yyz*_^(2)^ (*z*, ω_IR_) and the susceptibility χ̂_*yyz*_^(2)^ (*z*, ω_IR_) is that the former is a response function to general external fields, while the latter is a response function to electric fields. Ultimately, the experimental signal is determined by the integral over χ_*yyz*_^(2)^ (*z*,ω_IR_) and not χ̂_*yyz*_^(2)^ (*z*,ω_IR_). However, remaining parts of this work are formulated with respect to the susceptibility χ̂_*yyz*_^(2)^ (*z*,ω_IR_) and thus the difference needs to be clarified. Assuming classical nuclei motion, the imaginary part of the response function χ_*ijk*_^(2)″^(*z*,ω_IR_) is given by the fluctuation–dissipation relation shown in eq 5.
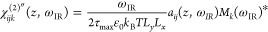
5Here *a*_*ij*_ (*z*,ω_IR_) is the frequency-dependent polarizability profile, *M*_*z*_ (ω_IR_)* is the complex conjugate of the frequency-dependent polarization of the entire system, *k*_*B*_ is the Boltzmann constant, *T* is the temperature, *τ*_max_ is the length of the trajectory, *L*_*x*_, *L*_*y*_ are the box dimensions in the plane of the interface. The trajectories are generated with the highly accurate MB-pol^86−88^ force field with classical nuclei dynamics using LAMMPS.^89^ The polarization is computed with the Thole-type polarizability model TTM-4F,^90^ included in MB-pol. The molecular polarizabilities are parametrized from single-molecule ab initio calculations on the CCSD(T)/aug-cc-pVTZ level, using Gaussian 16.^91^ Here we expand the molecular polarizability tensor in the molecular Eckart frame **α**_mol_ (*S*_1_,*S*_2_,*S*_3_) to first order in the symmetry coordinates *S*_1_,*S*_2_,*S*_3_.^92^ Accordingly, the time-dependent polarizability of the n^th^ molecule in the laboratory frame is given by eq 6
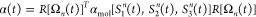
6where Ω_*n*_ (*t*) and S_1_^*n*^ (*t*),S_2_^*n*^ (*t*),S_3_^*n*^ (*t*) are the orientation of the Eckart frame and the symmetry coordinates of the n^th^ molecule, respectively, and ***R***[**Ω**_*n*_ (*t*)], is a rotation matrix. The influence of intermolecular interactions is accounted for by solving the self-consistent equation shown in eq 7 in each step iteratively.
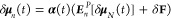
7Here δ**μ**_*n*_ is the induced dipole moment of the n^th^ molecule due to an external field δ***F*** and the field due to the induced dipoles on the other molecules *E*_*n*_^*P*^ [δ**μ**_*N*_ (*t*)]. The effective polarizability of the n^th^ molecule is then given by eq 8
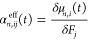
8where δμ_*n*,*i*_ (*t*) and δ*F*_j_ are the *i* and *j* components of the induced dipole moment and external field, respectively. Thus, the *z*-resolved polarizability profile of the system is given by eq 9
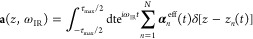
9where *Z*_*n*_(*t*) is the z-component of the center of mass of the n^th^ molecule. We bin all profiles with a bin size of 0.05 nm. In contrast to previous works,^71,93^ no cutoff or tapering is applied in the calculation of χ_*ijk*_^(2)^ (*z*,ω_IR_), instead, the intramolecular part of χ_*ijk*_^(2)^ (*z*,ω_IR_) is smoothed with a Hanning-window of length ω_Hann_^inter^ = 1.6 THz and the intermolecular one with a broader window length of ω_Hann_^inter^ = 14 THz. Furthermore, χ_*ijk*_^(2)^ (*z*,ω_IR_) is smoothed in space with a Gaussian window function with a standard deviation of σ = 0.038 nm. To generate the necessary amount of data, we generate 90 starting configurations from a 18 ns long simulation with the SPC/E^94^ force field using GROMACS^95^ with a time step of 2 fs in the *NVT* ensemble, implemented by the CSVR-thermostat^96^ with a relaxation time of 1 ps. For each of these 90 initial configurations we generate on average 0.24 ns long trajectories with the more expensive MB-Pol potential, using LAMMPS.^89^ In our analysis we discard the first 20 ps of each trajectory to give the system time to equilibrate to the new force field. Here we also use the CSVR-thermostat, but with a larger relaxation time of 5 ps and a smaller time step of 0.2 fs. We simulate 352 water molecules in a box with the dimensions *L*_*x*_ = *L*_*y*_ = 2 nm in the plane of the interface and *L*_*z*_ = 6 nm orthogonal to it. The slab thickness determined by the distances between the two Gibbs-dividing surfaces is 2.64 nm. Electrostatic interactions are computed using periodic boundary conditions with the particle mesh Ewald method, the electric field along the *z*-axis is corrected for periodicity effects.^83,97^ For the calculation of the effective polarizabilities a self-written Ewald-summation code is used.

## Data Availability

Raw data will be made available upon reasonable request by contacting the corresponding author.

## Supporting Information Available

Theory Behind Depth-Resolved SFG/DFG Spectroscopy, Absolute Zero Positions, Quadrupolar Contributions to the Water Response, The Nonresonant Response as a Phase Reference, and Frequency Dependence of the SFG-DFG Phase Difference (PDF)
