# Supplementary material for: How Thick is the Air–Water Interface?—A Direct Experimental Measurement of the Decay Length of the Interfacial Structural Anisotropy
Source: Langmuir. 2024 Aug 22;40(35):18760–72. doi: 10.1021/acs.langmuir.4c02571 (PMC11375779; doi:10.1021/acs.langmuir.4c02571)
Supplement: Supplementary file 1 — la4c02571_si_001.pdf [file la4c02571_si_001.pdf]

How Thick is the Air-Water Interface? - A Direct Experimental Measurement of the Decay Length of the Interfacial Structural Anisotropy

Supplementary Information

Alexander P. Fellows[1], Álvaro Díaz Duque[1], Vasileios Balos[2], Louis Lehmann[3], Roland R. Netz[3], Martin Wolf[1], and Martin Thämer[1]*

[1] Fritz-Haber-Institut der Max-Planck-Gesellschaft, Faradayweg 4-6, 14195, Berlin, Germany

[2] Instituto Madrileño de Estudios Avanzados en Nanociencia (IMDEA Nanociencia), 28049, Madrid, Spain

[3] Department of Physics, Freie Universität Berlin, Arnimallee 14, 14195, Berlin, Germany

* Corresponding author

thaemer@fhi-berlin.mpg.de

(tel.): +49 (0)30 8413 5220

Theory Behind Depth-Resolved SFG/DFG Spectroscopy

An in-depth description of the theory underlying the depth-resolved SFG technique can be found in references (*1*, *2*), only considering electric dipolar contributions. Here, the fundamental concepts of how it can be used to gain depth information are summarised and the theory is expanded to include electric quadrupolar sources in the following sections.

The measured SFG and DFG responses are governed by the effective second-order susceptibility, $\chi^{(2)}_{eff}$, which represents the spatially integrated response over the entire depth of the sample. For a signal sourcing from increasing depths, however, the input and output beams have longer path lengths and thus a propagation phase shift is introduced, ϕ_P. This phase is given by the product of depth and the z-component of the wavevector mismatch, Δk_z, as in Eq. 1.

$$\phi_P = \Delta k_z z \tag{1}$$

Clearly, the phase of the output signal for chromophores at increasing depths continuously cycles around the full 2π range. When these signals are integrated, however, the coherence length, $1/\Delta k_z$, defines the amplitude scaling and the output response is phase-shifted by a 90° propagation phase, as in Eq. 2 (on the assumption that the generation of output signals tends to zero as the depth tends to infinity).

$$\int_0^\infty dz\, \chi^{(2)} e^{i\Delta k_z z} = \left[\frac{1}{i\Delta k_z}\chi^{(2)} e^{i\Delta k_z z}\right]_0^\infty$$

$$= \frac{i}{\Delta k_z}\chi^{(2)} \tag{2}$$

The above expression in Eq. 2, however, assumes that there is no depth dependency to $\chi^{(2)}$. This assumption is thus not generally valid for dipolar sources since only anisotropic regions yield any signals and most bulk media are isotropic (all bulk liquids). Therefore, $\chi^{(2)}$ is generally bound to the interfacial region and thus can be described by a depth-dependent function that decays to zero with increasing distance from the interface. While the exact functional form of this depth-dependency is unknown, its decaying behaviour can be well-characterised by a decay length z' (healing depth) of an effective exponential decay function (see below for a more detailed discussion). Upon including this depth-dependency, the integration over depth can be explicitly solved, as in Eq. 3.

$$\int_0^\infty dz\, \chi^{(2)} e^{-\frac{z}{z'}} e^{i\Delta k_z z} = \frac{1}{\sqrt{\left(\frac{1}{z'}\right)^2 + (\Delta k_z)^2}}\chi^{(2)} e^{i\,\mathrm{atan}(\Delta k_z z')} \tag{3}$$

Clearly, the amplitude pre-factor scales as z' for short decay lengths, and tends to the coherence length as z' becomes large. Similarly, the phase-shift starts off approximately linear with z' but tends to ±90°, depending on the sign of Δk_z.

When probing resonances, the added propagation phase convolutes with the phase of the resonance, ϕ_R. Therefore, as the resonant phase is typically unknown, the propagation phase is generally inseparable when measuring the phase-resolved spectra from a single second-order response, as indicated in Eq. 4.

$$\phi_{eff} = \phi_R + \phi_P \tag{4}$$

Similarly, as the amplitudes of the resonances (magnitude of $\chi^{(2)}$) are also not generally known, accessing any depth information from the amplitude is also not possible.

By measuring both SFG and DFG phase-resolved responses, however, this convolution of resonant and depth information becomes separable. This is clearly the case as SFG and DFG involve different mixings of the input frequencies and thus must have different magnitude wavevector mismatches. For the input beams in this work, these values are ~0.02 nm^{-1} for SFG and ~0.014 nm^{-1} for DFG. It is important, however, to realise that the phase-resolved responses contain two halves of a complex conjugate pairing with negated frequency arguments, as described in Eq. 5 for SFG and Eq. 6 for DFG.

$$\chi^{(2)}_{eff}(\omega_2 + \omega_1, \omega_2, \omega_1) \;\; vs. \;\; \chi^{(2)}_{eff}(-\omega_2 - \omega_1, -\omega_2, -\omega_1) \tag{5}$$

$$\chi^{(2)}_{eff}(-\omega_2 + \omega_1, -\omega_2, \omega_1) \;\; vs. \;\; \chi^{(2)}_{eff}(\omega_2 - \omega_1, \omega_2, -\omega_1) \tag{6}$$

The sign of the resonant phase is dictated by the sign of the resonant frequency argument, taken to be ω_1 with no loss in generality. Therefore, by considering half of each phase-resolved response with the same sign of ω_1 (e.g., positive) the resonant information in each response is precisely equal. On the other hand, the sign of the wavevector mismatch is predominantly dictated by the signs of the two higher frequency arguments, which can clearly be seen to differ for the same sign of ω_1. This makes the effective phases of each response as in Eqs. 7 and 8.

$$\phi^{SFG}_{eff} = \phi_R + |\phi^{SFG}_P| \tag{7}$$

$$\phi^{DFG}_{eff} = \phi_R - |\phi^{DFG}_P| \tag{8}$$

Thus, by taking the phase difference of the two responses, the depth information becomes accessible. Assuming the exponential decay function, as above, this phase difference is given by Eq. 9.

$$\Delta\phi = \phi^{SFG}_{eff} - \phi^{DFG}_{eff} = \mathrm{atan}(|\Delta k^{SFG}_z| z') + \mathrm{atan}(|\Delta k^{DFG}_z| z') \tag{9}$$

From this, it is clear that the sensitivity to depth predominantly comes from the difference in sign of the two wavevector mismatches, not their difference in amplitude. This is clear as the phase difference yields an approximately linear increase over the first 10 nm range of decay lengths of ~1.9°nm^{-1}, only ~0.3°nm^{-1} of which comes from the magnitude mismatch. It is also worth noting the insensitivity of the amplitude ratio to decay length. As mentioned above, for relatively short decays, the amplitude pre-factor effectively scales as z' and is thus largely independent on Δk_z. Clearly, therefore, the amplitude ratio between DFG and SFG is effectively 1 for relatively short decay lengths. In fact, with the values for the wavevector mismatches here, it only yields ~1.01 for a 10 nm decay length, showing that it is effectively constant for the nanoscale decay lengths that could be expected for the structural anisotropy at an uncharged interface.

Demonstration of the Technique on Aqueous Interfaces

The feasibility of this technique to extract depth information from the phases of the responses has been previously demonstrated for model systems involving highly ordered self-assembled monolayers on solid surfaces. (*1*, *2*) To further show that its capabilities extend beyond these well-behaved systems to highly dynamic liquid interfaces, SFG and DFG spectra were recorded

from an aqueous interface covered in a negatively charged surfactant (dihexadecyl-hydrogenphosphate, DHP). At such charged aqueous interfaces, the excess interfacial charge generates a static electric field (E-field) which acts upon the molecular dipoles of water. This causes significant reorientation and thus generates an anisotropic environment, hence yielding a dipolar SFG signal. In low salinity conditions, the static E-field can extend for 10s or even 100s of nm away from the interface, and thus generate significant responses from depth, making this an excellent case for demonstrating the SFG/DFG technique.

Figure S1 shows a schematic of such an interface, highlighting the static E-field and induced reorientation favouring 'pointing-up' molecular dipoles. Also shown are the SFG and DFG spectra recorded in the C-H and O-H stretching region, split into their real and imaginary parts. On inspection of the spectra, both can clearly be seen to contain the sharp C-H resonances from the surfactant between 2800-3000 cm^{-1} as well as the broader O-H resonances from H_2O at higher frequencies. On comparing the spectra, however, it is clear that they do not have the same phase, demonstrating the effect of the substantial depth on the spectral line-shapes. This phase difference is emphasised in schematic Argand diagrams at four positions across the spectra, with the SFG and DFG responses being represented by a red and blue arrow, respectively. While the phase of each response clearly varies with frequency, as expected for vibrational resonances, the relative phase difference between the two responses that sources from the propagation depth is maintained. This clearly demonstrates that, through measurement of both responses, the resonant and depth-related phase contributions can be separated.

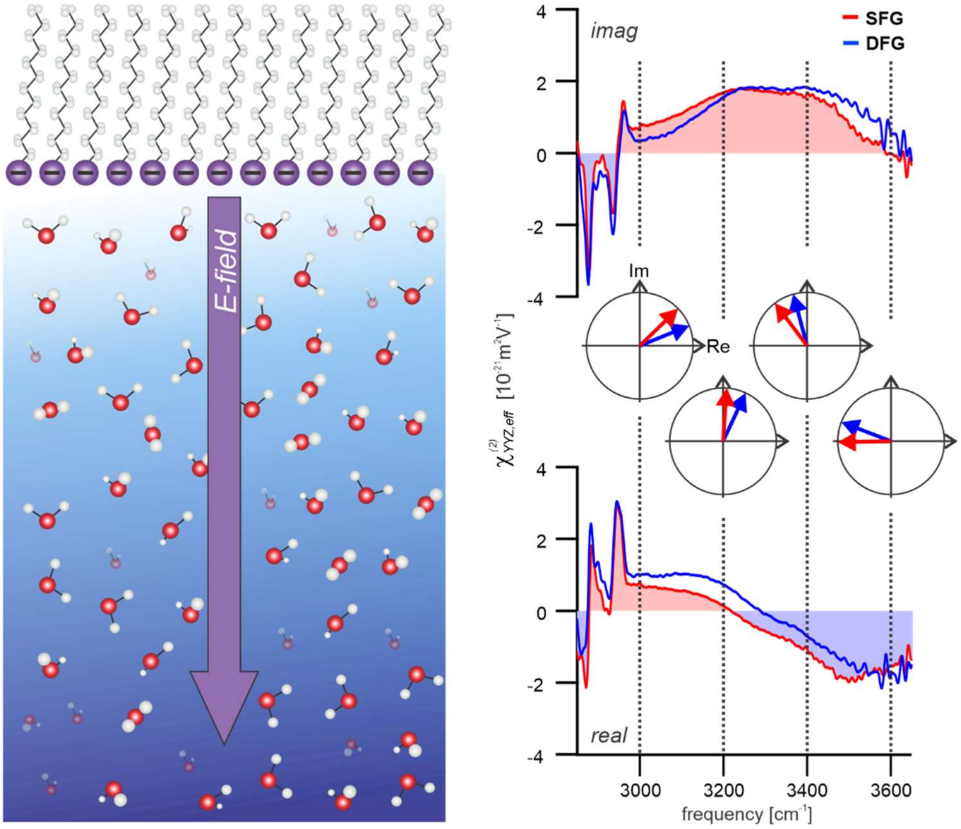

Figure S1: SFG and DFG responses of H_2O from an aqueous interface covered in a negatively charged surfactant, showing a schematic of the static electric field arising from the surface charge and induced reorientation of water with a preference for 'pointing up' molecular dipoles. Also shown are the real and imaginary parts of the recorded SFG and DFG spectra from the interface in absolute units and schematic representations of their phases at four points throughout the O-H stretching response.

Functional Form of the Depth-Dependent Decay

In the discussion above, the length-scale of the decaying depth-dependence of the second-order susceptibility is extracted by characterising the response by an effective exponential decay function. In reality, the anisotropic structure at the interface will have a complex functional form, likely showing molecular-level structure. Additionally, the effective (measured) susceptibility, representing the integrated response, not only includes the depth-dependence of the intrinsic susceptibility, but also that of the electric fields. As discussed in detail in the following sections, the effective susceptibility, $\chi_{eff}^{(2)}$, can be written as in Eq. 10, explicitly including the local field correction factors, $f(z)$, which account for the dielectric variation across the interface and thus for any depth-dependent variation in the field amplitudes.

$$\chi_{eff}^{(2)} = L^3 L^2 L^1 \int_0^\infty dz\, \chi_{ijk}^{(2)}(\omega_3, \omega_2, \omega_1, z) f^3(z) f^2(z) f^1(z) e^{-i\Delta k_z z} \tag{10}$$

These local field corrections hence represent a modulation of the signal amplitudes within the first few Ångströms, thus slightly altering the effective functional form of the decay. Since the measurements are both spatially (in-plane) and temporally averaged any molecular-scale distance modulations to the functional form are smoothed out. Overall, therefore, describing the effective functional form with an exponential decay function represents a suitable method for extracting its length-scale.

Inclusion of an Isotropic Contribution

In the above treatment, it was assumed that only electric dipolar sources contribute, thus limiting the integration to the structurally anisotropic region in proximity to the interface. As shown in the later sections, however, quadrupolar sources are not restricted to structurally anisotropic environments and thus can source throughout the entire bulk. Specifically, two different types of quadrupolar contributions can also be included, one arising from the dielectric anisotropy at the interface (interfacial quadrupolar), and one from the isotropic bulk (bulk quadrupolar). The former contribution can be treated just as the dipolar contributions, only now accounting for the dielectric anisotropy rather than the structural anisotropy. This makes the dipolar and interfacial quadrupolar contributions effectively inseparable from SFG and DFG measurements. In contrast, the bulk quadrupolar contribution is inherently phase shifted by 90° and multiplied by the wavevector of the upconversion beam (assuming SSP polarisation, see later). These 90°-shifted contributions are then integrated over effectivity infinite depth with a depth-independent susceptibility. The overall response for SFG/DFG can thus be written as the sum of a decaying anisotropic contribution (dipolar and interfacial quadrupolar) and a constant isotropic term (bulk quadrupolar), as in Eq. 11.

$$\begin{aligned}\chi_{eff}^{(2)SFG,DFG} &= \int_0^\infty dz\, \chi_{aniso}^{(2)} e^{-\frac{z}{z'}} e^{i\Delta k_z^{SFG,DFG} z} + \int_0^\infty dz\, i k_2^{SFG,DFG} \chi_{iso}^{(2)} e^{i\Delta k_z^{SFG,DFG} z} \\ &= \frac{1}{\sqrt{\left(\frac{1}{z'}\right)^2 + \left(\Delta k_z^{SFG,DFG}\right)^2}} \chi_{aniso}^{(2)} e^{i\,\mathrm{atan}\left(\Delta k_z^{SFG,DFG} z'\right)} - \frac{k_2^{SFG,DFG}}{\Delta k_z^{SFG,DFG}} \chi_{iso}^{(2)}\end{aligned} \tag{11}$$

From this, it is clear that the anisotropic contribution yields a phase difference between SFG and DFG but essentially no amplitude difference (assuming a decay length <10 nm). In contrast, the isotropic contribution yields no phase difference but clearly different amplitudes. This is

evident since, whilst SFG and DFG use the same upconversion field, the responses with equal resonant phases (i.e., positive ω_1, see Eqs. 5 and 6) use different halves of its complex conjugate pairing i.e., have different signs for k_2. This sign difference is equally reflected in their wavevector mismatches and thus the only difference between the isotropic contribution for the two responses is from their respective coherence lengths (i.e., from the magnitude difference of their wavevector mismatches). Therefore, by combining the two contributions, namely anisotropic and isotropic, the amplitude ratio of the effective responses will solely speak to the relative proportion of the isotropic component, and the phase difference will be approximately linear with decay length (over a 10 nm range), but with a gradient that also depends on the isotropic proportion. This dependency is shown in Figure 2 in the main text. Through measuring both responses, each of these parameters can thus be determined.

Even for systems with longer anisotropic decay lengths, beyond which the assumption of equal amplitudes becomes poor, the measurement of both SFG and DFG still allows for a full determination of the relative isotropic proportion and decay length of the anisotropy. This is clear as the phase difference and amplitude ratio for the anisotropic contribution are inherently linked and thus should both reflect the exact decay length. A modelled plot of both parameters is shown in Figure S2 for anisotropic decay lengths up to 100 nm and relative isotropic contributions (ratios to the anisotropic component) varying from +1.0 to -1.0. From this, it is clear that an experimentally determined pair of values for the two parameters can be used to directly access both quantities, with an example being highlighted for an observed phase difference of 60° and an amplitude ratio of 1.1.

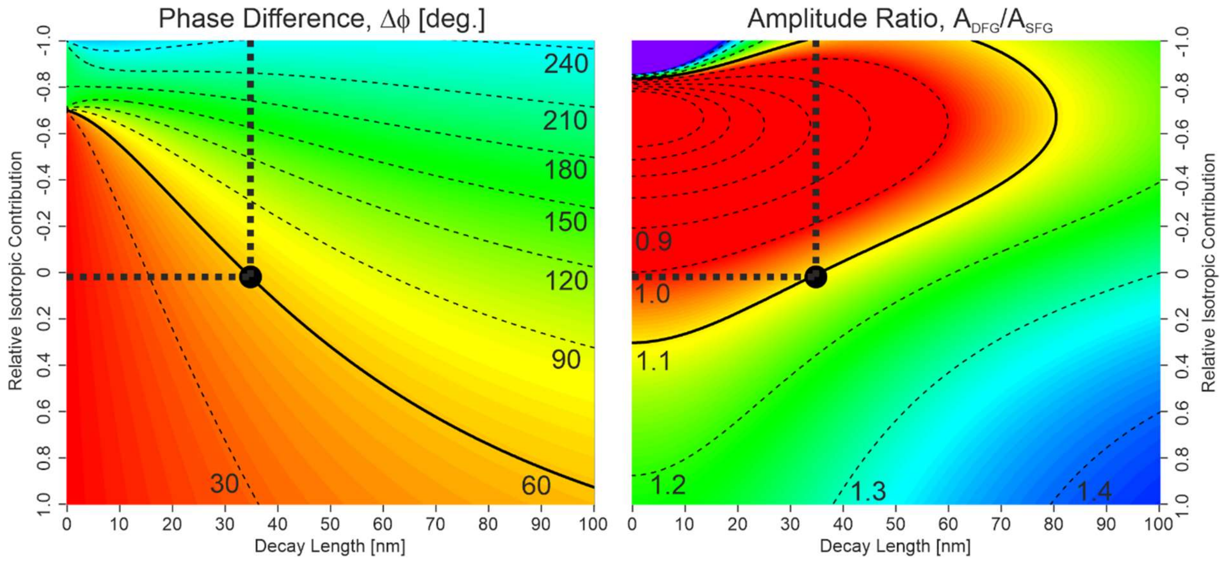

Figure S2: Modelled plots of the SFG-DFG phase difference and amplitude ratio as a function of both the anisotropic decay length and the relative isotropic contribution. Contour lines are also included for better clarity and an example extraction of the desired quantities is shown for a phase difference of 60° and amplitude ratio of 1.1.

Absolute zero positions

In the above theoretical description of the depth-resolved SFG-DFG spectroscopy it is clear that the propagation phase can be extracted, but it is not immediately obvious what the z=0 position is which yields no additional phase. However, given that the signals generated from a sample surface are heterodyned with SFG and DFG local oscillators (LO), these reference signals provide the phase reference for each pulse. As such, since they are linearly reflected from the

same surface, the reference depth must be the plane of linear reflection (PLR). This effective depth is hence governed by the gradient in the dielectric function, and thus exists very close to the top-most surface region.

As noted above, however, the absolute phases are initially determined by referencing to a measurement from z-cut quartz, assuming it to be an entirely bulk dipolar signal, thus with a phase of ±90°. However, since quartz is known to deviate slightly from this phase owing to its structural changes at the surface(*3*), the absolute phases are then corrected by a SFG-DFG measurement from a self-assembled monolayer of OTS on fused silica. Specifically, the methyl resonances from the OTS film are phase-rotated such that they are exactly equal in SFG and DFG. This hence sets the zero-depth position to be the position of the methyl groups, not the PLR i.e., z=0 is effectively set to the on-set of density change from air into the sample. Therefore, given that the change in both density and dielectric function is relatively similar across the air-water and air-SAM-FS interfaces, this depth correction factor is expected to also essentially move the z=0 position from the PLR to the top-most water layer which corresponds to the on-set of the density change.

For the simulation results, the boundary plane is instead defined as the Gibbs diving surface (GDS) which represents the midpoint of the density change between the two phases. Therefore, the simulated depths will be offset to the experimental depth by approximately half the width of the density change profile. As the density changes over ~3-5Å, the simulated depths are thus expected to be offset by about -2Å. By comparing the data in Figure 5 in the main text, it is clear that the simulations do indeed predict the on-set of the SFG signals to occur at ~-2Å.

The Effect of Capillary Waves

In the discussion above the interface is taken to be perfectly flat. In reality, however, capillary waves are ever-present and modulate the z-coordinates of the interface by several Ångströms. While these capillary waves obviously smear-out the interfacial region, they are unlikely to affect the result of SFG/DFG measurements. The reason for this is associated with the way in which the intrinsic zero-position is spectroscopically defined using the linear reflection of the LO. The measured quantity is then the spatial distance between the planes of linear reflection and the source of the non-linear signal, both of which are similarly modulated by capillary waves. The resulting effective z-positions of the planes thus still represent the thickness of the structurally anisotropic interfacial region as deviations in absolute z-positions due to capillary waves should be largely cancelled by taking their difference.

Quadrupolar Contributions to the Water Response

From the spectra shown in Figure 3(a) in the main text, there is a clear offset in amplitude between SFG and DFG in the real part of the measured response, but no distinct difference in imaginary part. Within the electric dipole approximation, the intrinsic SFG and DFG responses are equal in the absence of dispersion (as shown by the permutation symmetry relations discussed below). Since dispersion is shown to be insignificant in Figure 4(b), any discrepancy between SFG and DFG can only arise from the integration of the (potentially depth-dependent) intrinsic responses. Whilst a depth in the anisotropy does lead to a larger DFG response cf. SFG, it also introduces a phase difference between SFG and DFG for dipolar sources.(*1*, *2*) However, the spectra in Figure 3(a) clearly show that there is negligible phase difference between SFG and DFG, indicating that the source of this discrepancy cannot arise from depth based on the electric dipole mechanism. Instead, however, one must look beyond the electric dipole contributions to explain this observation.

In the following discussion, the second-order responses are expanded beyond the electric dipole contribution to include quadrupolar mechanisms, broadly following the theoretical treatment and notation given by Morita(*4*). Initially, the terms arising from these mechanisms are derived and their contributions to SFG and DFG pathways compared. In this context, there are two important scenarios to consider. The first is the comparison between SFG and DFG responses produced simultaneously from the same experiment and thus using the same input beams (but therefore having different output frequencies). In this scenario, there is no inherent permutation symmetry in the intrinsic dipolar susceptibilities for both outputs. The second scenario compares the SFG and DFG responses involving the same three beams, where the DFG is produced by using an upconversion beam at the SFG frequency (labelled DFG' as in the main text). Whilst permutation symmetry for the dipolar response hold in this comparison, the two responses must necessarily arise from separate measurements.(*1*)

Once the expressions behind these comparisons are derived in each scenario, we then move to analyse the measured non-resonant response from H_2O. From the amplitude ratios between the SFG and both DFG responses, it is determined that the bulk quadrupolar contribution appears to be the source of the observed discrepancy. Thereafter, the implications of these results are discussed.

Expansion to the Quadrupolar Terms

By analogy to the linear polarisability, the second-order susceptibility can be expanded in a Taylor series based on its spatial coordinates, as in Eq. 12.

$$\chi^{(2)} = \chi_0^{(2)} + \left(\frac{\partial \chi^{(2)}}{\partial r}\right)_{r_0} (r - r_0) + \cdots \tag{12}$$

This expansion can then be input into the expression for the second-order polarisation, as in Eq. 13.

$$\begin{aligned} P^{(2)} &= \varepsilon_0 \chi^{(2)} \colon E_1 E_2 \\ &= \varepsilon_0 \chi_0^{(2)} \colon E_1 E_2 + \varepsilon_0 \left(\frac{\partial \chi^{(2)}}{\partial r}\right)_{r_0} (r - r_0) \colon E_1 E_2 + \cdots \end{aligned} \tag{13}$$

Therefore, by means of the product rule, the second term can be rewritten as in Eq. 14.

$$\begin{aligned} \left(\frac{\partial \chi^{(2)}}{\partial r}\right)_{r_0} (r - r_0) \colon E_1 E_2 &= \frac{\partial}{\partial r}\left(\chi^{(2)}(r - r_0) \colon E_1 E_2\right) - \chi^{(2)} \colon E_1 E_2 \\ &-\chi^{(2)}(r - r_0) \colon \frac{\partial E_1}{\partial r} E_2 - \chi^{(2)}(r - r_0) \colon E_1 \frac{\partial E_2}{\partial r} \end{aligned} \tag{14}$$

The four terms on the right side of Eq. 14 each describe different mechanistic pathways towards generation of a second-order polarisation. Specifically, the first term, involving the derivative of the whole product, describes the dipole produced from the gradient of an induced quadrupole. By contrast, the second term simply describes the dipole produced by the electric dipole mechanism. Finally, the remaining two terms describe dipoles produced using both an electric field and the gradient of another electric field, and thus must be quadrupolar in origin (neglecting magnetic dipole mechanisms). Clearly, the three terms based on quadrupolar processes are described by a rank four tensors, and are thus non-cancelling in isotropic environments. Based on this description of the different processes, the four terms can be written

with unique susceptibilities labelled as Q, D0, D1, and D2, respectively, indicating the mechanism of generation. The second-order polarisation can thus be written as in Eq. 15, assuming no inherent polarisation.

$$P^{(2)} = \varepsilon_0 \chi^{(2),D0}\colon E_1 E_2 + \varepsilon_0 \chi^{(2),D1}\colon \frac{\partial E_1}{\partial r} E_2 + \varepsilon_0 \chi^{(2),D2}\colon E_1 \frac{\partial E_2}{\partial r} - \varepsilon_0 \frac{\partial}{\partial r}\left(\chi^{(2),Q}\colon E_1 E_2\right) \quad (15)$$

In order to describe the full measured response, one must then integrate these contributions over infinite depth through the sample. The effective second-order susceptibility is then given by Eq. 16, where the generation processes are driven using local fields, $\tilde{E}_i^n$, which account for both Fresnel factors, L_{ii}^n, and depth-dependent local field factors, f_i^n, as in Eq. 17.

$$\begin{aligned}\chi_{ijk}^{(2),eff} E_j^2 E_k^1 = \sum_{jks}^{x,y,z} \int_{-\infty}^{\infty} dz\, f_i^3 L_{ii}^3 \Big[&\chi_{ijk}^{(2),D0}(\omega_3,\omega_2,\omega_1,z)\, f_k^1 L_{kk}^1 E_k^1 f_j^2 L_{jj}^2 E_j^2 \\ &+ \chi_{ijks}^{(2),D1}(\omega_3,\omega_2,\omega_1,z) \frac{\partial f_k^1 L_{kk}^1 E_k^1}{\partial s} f_j^2 L_{jj}^2 E_j^2 \\ &+ \chi_{ijks}^{(2),D2}(\omega_3,\omega_2,\omega_1,z) f_k^1 L_{kk}^1 E_k^1 \frac{\partial f_j^2 L_{jj}^2 E_j^2}{\partial s} \\ &- \frac{\partial}{\partial s}\left(\chi_{ijks}^{(2),Q}(\omega_3,\omega_2,\omega_1,z) f_k^1 L_{kk}^1 E_k^1 f_j^2 L_{jj}^2 E_j^2\right)\Big] e^{-ik_z^3 z} \end{aligned} \quad (16)$$

$$\tilde{E}_i^n(z) = f_i^n(z) L_{ii}^n E_i^n \quad (17)$$

The integral in Eq. 16 may then be broken down based on knowledge about the spatial variation and symmetry properties of the different parameters. Firstly, the Fresnel factors are spatially invariant and purely related to the beam polarisation, experimental geometry, and dielectric constants (which give them frequency dependence). By contrast, both the local field factors and the input fields have spatial variations. The local field factors only vary with depth very close to the interface due to the continuous change in the dielectric function between the incident and bulk media. On the other hand, the incident fields only have spatial variations due to their oscillatory nature, thus depending on their wavevectors, and exist throughout the entire integral range. This allows the integral to be broken up into different contributions, namely those isolated to the interfacial range ($z = 0 \rightarrow z_0$) and those covering the entire sample ($z = 0 \rightarrow \infty$), as in Eq. 18.

$$\chi_{ijk}^{(2),eff} E_j^2 E_k^1 = \sum_{jks}^{x,y,z} L_{ii}^3 L_{jj}^2 L_{kk}^1 \left[\int_0^{z_0} dz\, \chi_{ijk}^{(2),D0}(\omega_3,\omega_2,\omega_1,z) f_k^1(z) f_j^2(z) f_i^3(z) E_k^1 E_j^2 e^{-ik_z^3 z} \right. \quad (18a)$$

$$+ \int_0^{z_0} dz\, \chi_{ijkz}^{(2),D1}(\omega_3,\omega_2,\omega_1,z) \frac{\partial f_k^1(z)}{\partial z} f_j^2(z) f_i^3(z) E_k^1 E_j^2 e^{-ik_z^3 z} \quad (18b)$$

$$+ \int_0^{\infty} dz\, \chi_{ijks}^{(2),D1}(\omega_3,\omega_2,\omega_1,z) f_k^1(z) f_j^2(z) f_i^3(z) \frac{\partial E_k^1}{\partial s} E_j^2 e^{-ik_z^3 z} \quad (18c)$$

$$+ \int_0^{z_0} dz\, \chi_{ijkz}^{(2),D2}(\omega_3,\omega_2,\omega_1,z) f_k^1(z) \frac{\partial f_j^2(z)}{\partial z} f_i^3(z) E_k^1 E_j^2 e^{-ik_z^3 z} \quad (18d)$$

$$+\int_0^{\infty} dz\, \chi_{ijks}^{(2),D2}(\omega_3,\omega_2,\omega_1,z) f_k^1(z) f_j^2(z) f_i^3(z) E_k^1 \frac{\partial E_j^2}{\partial s} e^{-ik_z^3 z} \quad (18e)$$

$$-\int_0^{z_0} dz\, f_i^3(z) \frac{\partial}{\partial z}\left(\chi_{ijkz}^{(2),Q}(\omega_3,\omega_2,\omega_1,z) f_k^1(z) f_j^2(z)\right) E_k^1 E_j^2 e^{-ik_z^3 z} \quad (18f)$$

$$\left.-\int_0^{\infty} dz\, \chi_{ijks}^{(2),Q}(\omega_3,\omega_2,\omega_1,z) f_k^1(z) f_j^2(z) f_i^3(z) \frac{\partial}{\partial s}\left(E_k^1 E_j^2\right) e^{-ik_z^3 z}\right] \quad (18g)$$

For the terms involving integration over the full depth (18c, e, and g), any variation in both the local field factors and susceptibilities can be neglected as the vast majority of the signal arises from the isotropic bulk. Since the z_0 depth is defined to be the point at which any anisotropy vanishes, the bulk quantities can be taken as those evaluated at this depth. On the other hand, for the interfacial terms, any variation in the oscillatory field contributions can also be neglected on the assumption that z_0 is much smaller than the wavelength. This allows Eq. 18 to be simplified as in Eq. 19, having explicitly included the derivatives of the oscillatory fields in the bulk terms.

$$\chi_{ijk}^{(2),eff} E_j^2 E_k^1 = \sum_{jks}^{x,y,z} L_{ii}^3 L_{jj}^2 L_{kk}^1 \left[\int_0^{z_0} dz \left(\chi_{ijk}^{(2),D0}(\omega_3,\omega_2,\omega_1,z) f_k^1(z) f_j^2(z) f_i^3(z)\right)\right. \quad (19a)$$

$$+\int_0^{z_0} dz\, \chi_{ijkz}^{(2),D1}(\omega_3,\omega_2,\omega_1,z) \frac{\partial f_k^1(z)}{\partial z} f_j^2(z) f_i^3(z) \quad (19b)$$

$$+\chi_{ijks}^{(2),D1}(\omega_3,\omega_2,\omega_1,z_0) f_k^1(z_0) f_j^2(z_0) f_i^3(z_0) \int_0^{\infty} dz\, ik_s^1 e^{i(k_z^1+k_z^2-k_z^3)z} \quad (19c)$$

$$+\int_0^{z_0} dz\, \chi_{ijkz}^{(2),D2}(\omega_3,\omega_2,\omega_1,z) f_k^1(z) \frac{\partial f_j^2(z)}{\partial z} f_i^3(z) \quad (19d)$$

$$+\chi_{ijks}^{(2),D2}(\omega_3,\omega_2,\omega_1,z_0) f_k^1(z_0) f_j^2(z_0) f_i^3(z_0) \int_0^{\infty} dz\, ik_s^2 e^{i(k_z^1+k_z^2-k_z^3)z} \quad (19e)$$

$$-\int_0^{z_0} dz\, f_i^3(z) \frac{\partial}{\partial z}\left(\chi_{ijkz}^{(2),Q}(\omega_3,\omega_2,\omega_1,z) f_k^1(z) f_j^2(z)\right) \quad (19f)$$

$$\left.-\chi_{ijks}^{(2),Q}(\omega_3,\omega_2,\omega_1,z_0) f_k^1(z_0) f_j^2(z_0) f_i^3(z_0) \int_0^{\infty} dz\, i(k_s^1+k_s^2) e^{i(k_z^1+k_z^2-k_z^3)z}\right] |E_k^1||E_j^2| \quad (19g)$$

The three bulk terms can then be directly evaluated, yielding the terms 20c, e, and g. Similarly, by integration by parts, term 19f can be expanded into two contributions, as terms 20f.1 and 20f.2 in Eq. 20.

$$\chi_{ijk}^{(2),eff} E_j^2 E_k^1 = \sum_{jks}^{x,y,z} L_{ii}^3 L_{jj}^2 L_{kk}^1 \left[\int_0^{z_0} dz \left(\chi_{ijk}^{(2),D0}(\omega_3, \omega_2, \omega_1, z) f_k^1(z) f_j^2(z) f_i^3(z) \right) \right. \quad (20a)$$

$$+ \int_0^{z_0} dz\, \chi_{ijkz}^{(2),D1}(\omega_3, \omega_2, \omega_1, z) \frac{\partial f_k^1(z)}{\partial z} f_j^2(z) f_i^3(z) \quad (20b)$$

$$-\chi_{ijks}^{(2),D1}(\omega_3, \omega_2, \omega_1, z_0) f_k^1(z_0) f_j^2(z_0) f_i^3(z_0) \frac{k_s^1}{k_z^1 + k_z^2 - k_z^3} \quad (20c)$$

$$+ \int_0^{z_0} dz\, \chi_{ijkz}^{(2),D2}(\omega_3, \omega_2, \omega_1, z) f_k^1(z) \frac{\partial f_j^2(z)}{\partial z} f_i^3(z) \quad (20d)$$

$$-\chi_{ijks}^{(2),D2}(\omega_3, \omega_2, \omega_1, z_0) f_k^1(z_0) f_j^2(z_0) f_i^3(z_0) \frac{k_s^2}{k_z^1 + k_z^2 - k_z^3} \quad (20e)$$

$$+ \int_0^{z_0} dz \frac{\partial f_i^3(z)}{\partial z} \chi_{ijkz}^{(2),Q}(\omega_3, \omega_2, \omega_1, z) f_k^1(z) f_j^2(z) \quad (20f.1)$$

$$-\chi_{ijkz}^{(2),Q}(\omega_3, \omega_2, \omega_1, z_0) f_k^1(z_0) f_j^2(z_0) f_i^3(z_0) \quad (20f.2)$$

$$\left. +\chi_{ijks}^{(2),Q}(\omega_3, \omega_2, \omega_1, z_0) f_k^1(z_0) f_j^2(z_0) f_i^3(z_0) \frac{k_s^1 + k_s^2}{k_z^1 + k_z^2 - k_z^3} \right] |E_k^1||E_j^2| \quad (20g)$$

These terms can be grouped together based on the mechanism of their dipole induction and source location, with 20a being the interfacial dipole term (ID), 20b, 20d, and 20f.1 being interfacial quadrupole terms (IQ), 20c, 20e, and 20g being bulk quadrupole terms (QB), and 20f.2 being an interfacial quadrupolar contribution but with bulk-like properties (IQB). The effective susceptibility can thus be written as a sum of these contributions, as in Eq. 21.

$$\chi_{eff}^{(2)} = \chi^{(2)ID} + \chi^{(2)IQ} + \chi^{(2)IQB} + \chi^{(2)QB} \quad (21)$$

Comparing SFG and DFG Responses

If we start by comparing the SFG and DFG responses that involve the same three frequencies (SFG and DFG'), and specifically only talking one half of the phase-resolved response involving a positive ω_1 argument, one must consider the permutation symmetry between the intrinsic susceptibilities. By limiting our considerations to processes that start in the ground vibrational state, then the SFG and DFG' processes are contributed to by two and three pairs of correlation functions (pathways), with each pair being complex conjugates that just represent a frequency negation.(*1*) Specifically, by taking only positive ω_1 as above, the SFG and DFG' susceptibilities can be written as in Eqs. 22 and 23, where a, b, and c are used in the numerators to represent either μ or q (where the additional tensor dimension, s, is associated only with q). This allows us to consider either dipolar or quadrupolar transitions under any of the aforementioned mechanisms. (Note that the DFG' response deliberately has the 'i' and 'j' indices reversed.)

$$\tilde{\chi}^{(2)}_{ijk(s)}(\omega_2+\omega_1,\omega_2,\omega_1)=\frac{N}{2\varepsilon_0\hbar^2}\sum_{qr}\frac{a^{(s)i}_{gr}b^{(s)j}_{rq}c^{(s)k}_{qg}}{(\omega_1-\omega_{qg}+i\Gamma_{qg})(\omega_2+\omega_1-\omega_{rg}+i\Gamma_{rg})} \tag{22.1}$$

$$+\frac{a^{(s)i}_{gr}b^{(s)j}_{qg}c^{(s)k}_{rq}}{(\omega_2-\omega_{qg}+i\Gamma_{qg})(\omega_2+\omega_1-\omega_{rg}+i\Gamma_{rg})} \tag{22.2}$$

$$\tilde{\chi}^{(2)}_{jik(s)}(-\omega_2,-\omega_2-\omega_1,\omega_1)=\frac{N}{2\varepsilon_0\hbar^2}\sum_{qr}\frac{a^{(s)i}_{gr}b^{(s)j}_{rq}c^{(s)k}_{qg}}{(\omega_1-\omega_{qg}+i\Gamma_{qg})(\omega_2-\omega_{rq}-i\Gamma_{rq})} \tag{23.1}$$

$$-\frac{a^{(s)i}_{gr}b^{(s)j}_{rq}c^{(s)k}_{qg}}{(\omega_2+\omega_1-\omega_{rg}-i\Gamma_{rg})(\omega_2-\omega_{rq}-i\Gamma_{rq})} \tag{23.2}$$

$$+\frac{a^{(s)i}_{gr}b^{(s)j}_{qg}c^{(s)k}_{rq}}{(\omega_2+\omega_1-\omega_{rg}-i\Gamma_{rg})(\omega_2-\omega_{qg}-i\Gamma_{qg})} \tag{23.3}$$

The DFG' terms 23.1 and 23.2 can be trivially factorised and combined to yield Eq. 24.

$$\tilde{\chi}^{(2)}_{jik(s)}(-\omega_2,-\omega_2-\omega_1,\omega_1)=$$

$$\frac{N}{2\varepsilon_0\hbar^2}\sum_{qr}\frac{a^{(s)i}_{gr}b^{(s)j}_{rq}c^{(s)k}_{qg}}{(\omega_2-\omega_{rq}-i\Gamma_{rq})}\left\{\frac{\omega_2-\omega_{rq}-i(\Gamma_{rg}+\Gamma_{qg})}{(\omega_1-\omega_{qg}+i\Gamma_{qg})(\omega_2+\omega_1-\omega_{rg}-i\Gamma_{rg})}\right\} \tag{24.1}$$

$$+\frac{a^{(s)i}_{gr}b^{(s)j}_{qg}c^{(s)k}_{rq}}{(\omega_2+\omega_1-\omega_{rg}-i\Gamma_{rg})(\omega_2-\omega_{qg}-i\Gamma_{qg})} \tag{24.2}$$

Whilst this is not the same as the analogous expression for SFG in Eq. 22, when the two higher frequency arguments are off-resonant, the two expressions clearly converge, independent on the transition mechanisms. This shows that the permutation symmetry holds for all of the aforementioned intrinsic susceptibilities (D0, D1, D2, and Q) as long as $i=j$, which is necessarily the case when using the SSP polarisation combination, as used here.

Interfacial Dipole Contribution (ID)

Firstly, the dipole responses from SFG and DFG' are considered, being given in Eqs. 25 and 26, respectively, exploiting the intrinsic permutation symmetry relation between the two susceptibilities along with the symmetry of the local field factors on negation of the input frequencies. Clearly, the overall SFG and DFG' responses from this mechanism are equal.

$$\chi^{(2)ID}_{SSP}(\omega_2+\omega_1,\omega_2,\omega_1)=$$

$$\sum_{k}^{x,z}\int_0^{z_0}dz\,\chi^{(2),D0}_{yyk}(\omega_2+\omega_1,\omega_2,\omega_1,z)f_k(\omega_1,z)f_y(\omega_2,z)f_y(\omega_2+\omega_1,z) \tag{25}$$

$$\chi^{(2)ID}_{SSP}(-\omega_2,-\omega_2-\omega_1,\omega_1)=$$

$$\sum_{k}^{x,z}\int_0^{z_0}dz\,\chi^{(2),D0}_{yyk}(\omega_2+\omega_1,\omega_2,\omega_1,z)f_k(\omega_1,z)f_y(\omega_2,z)f_y(\omega_2+\omega_1,z) \tag{26}$$

Interfacial Quadrupolar Contribution (IQ)

Next, if we consider the interfacial quadrupolar contributions to the same two responses, they are given by Eqs. 27 and 28.

$$\chi_{SSP}^{(2)IQ}(\omega_2+\omega_1,\omega_2,\omega_1) = \sum_k^{x,z}\int_0^{z_0} dz \left[\chi_{yykz}^{(2),D1}(\omega_2+\omega_1,\omega_2,\omega_1,z)\frac{\partial f_k(\omega_1,z)}{\partial z} f_y(\omega_2,z) f_y(\omega_2+\omega_1,z) + \chi_{yykz}^{(2),D2}(\omega_2+\omega_1,\omega_2,\omega_1,z) f_k(\omega_1,z)\frac{\partial f_y(\omega_2,z)}{\partial z} f_y(\omega_2+\omega_1,z) + \chi_{yykz}^{(2),Q}(\omega_2+\omega_1,\omega_2,\omega_1,z) f_k(\omega_1,z) f_y(\omega_2,z)\frac{\partial f_y(\omega_2+\omega_1,z)}{\partial z}\right] \quad (27)$$

$$\chi_{SSP}^{(2)IQ}(-\omega_2,-\omega_2-\omega_1,\omega_1) = \sum_k^{x,z}\int_0^{z_0} dz \left[\chi_{yykz}^{(2),D1}(\omega_2+\omega_1,\omega_2,\omega_1,z)\frac{\partial f_k(\omega_1,z)}{\partial z} f_y(\omega_2,z) f_y(\omega_2+\omega_1,z) + \chi_{yykz}^{(2),D2}(\omega_2+\omega_1,\omega_2,\omega_1,z) f_k(\omega_1,z)\frac{\partial f_y(\omega_2+\omega_1,z)}{\partial z} f_y(\omega_2,z) + \chi_{yykz}^{(2),Q}(\omega_2+\omega_1,\omega_2,\omega_1,z) f_k(\omega_1,z) f_y(\omega_2+\omega_1,z)\frac{\partial f_y(\omega_2,z)}{\partial z}\right] \quad (28)$$

At this point, however, it is clear that the interfacial dipole responses for SFG and DFG' are not intrinsically the same. Whilst the D1 contribution is equivalent to both, the D2 and Q terms have swapped their local field factor modulation functions, with these not generally being equal, as in Eq. 29.

$$\frac{\partial f_y(\omega_2+\omega_1,z)}{\partial z} f_y(\omega_2,z) \neq f_y(\omega_2+\omega_1,z)\frac{\partial f_y(\omega_2,z)}{\partial z} \quad (29)$$

Nevertheless, as these two frequencies are off-resonant and typically close in frequency (with ω_1 being in the mid-IR), any dispersion in their dielectric functions is likely small and thus, to a good approximation, the inequality in Eq. 29 can be taken to be equal.

By considering the simultaneously generated DFG response (with the same upconversion but different output frequency as the SFG), similar expressions can be generated only now without permutation symmetry between the intrinsic susceptibilities, as shown in Eq. 30.

$$\chi_{SSP}^{(2)IQ}(-\omega_2+\omega_1,-\omega_2,\omega_1) = \sum_k^{x,z}\int_0^{z_0} dz \left[\chi_{yykz}^{(2),D1}(-\omega_2+\omega_1,-\omega_2,\omega_1,z)\frac{\partial f_k(\omega_1,z)}{\partial z} f_y(\omega_2,z) f_y(\omega_2-\omega_1,z) + \chi_{yykz}^{(2),D2}(-\omega_2+\omega_1,-\omega_2,\omega_1,z) f_k(\omega_1,z)\frac{\partial f_y(\omega_2,z)}{\partial z} f_y(\omega_2-\omega_1,z) + \chi_{yykz}^{(2),Q}(-\omega_2+\omega_1,-\omega_2,\omega_1,z) f_k(\omega_1,z) f_y(\omega_2,z)\frac{\partial f_y(\omega_2-\omega_1,z)}{\partial z}\right] \quad (30)$$

Now, equality between the SFG and DFG only exists on the assumption of no dispersion in the high frequency range, both in the local field factors and the intrinsic susceptibilities. Both this

assumption and the one above for the permutation-symmetric response are, however, somewhat experimentally validated in Figure 4(b) in the main text where the two different DFG responses (DFG and DFG') are compared, showing only minute differences.

Interfacial Quadrupolar Contribution with Bulk-like Properties (IQB)

The comparison between SFG and DFG' for the interfacial quadrupolar contribution with bulk-like properties is very straightforward, as it only constitutes a single term with no derivatives of local field factors. The two contributions can be written as in Eqs. 31 and 32, again including the permutation symmetry for DFG'.

$$\chi_{SSP}^{(2)IQB}(\omega_2+\omega_1,\omega_2,\omega_1) = -\sum_{k}^{x,z}\chi_{yykz}^{(2),Q}(\omega_2+\omega_1,\omega_2,\omega_1,z_0)f_k(\omega_1,z_0)f_y(\omega_2,z_0)f_y(\omega_2+\omega_1,z_0) \tag{31}$$

$$\chi_{SSP}^{(2)IQB}(-\omega_2,-\omega_2-\omega_1,\omega_1) = -\sum_{k}^{x,z}\chi_{yykz}^{(2),Q}(\omega_2+\omega_1,\omega_2,\omega_1,z_0)f_k(\omega_1,z_0)f_y(\omega_2,z_0)f_y(\omega_2+\omega_1,z_0) \tag{32}$$

This shows that the two responses are identical. If we then consider the simultaneous DFG response, a similar expression to Eq. 32 can be generated, only with a frequency shift where equality is only reached with no dispersion.

Bulk Quadrupolar Contribution (QB)

For the bulk quadrupolar contributions, the SFG and permutation-symmetric DFG' responses are given by Eqs. 33 and 34.

$$\chi_{SSP}^{(2)QB}(\omega_2+\omega_1,\omega_2,\omega_1) = \sum_{s}^{x,y,z}\sum_{k}^{x,z}\frac{f_k(\omega_1,z_0)f_y(\omega_2,z_0)f_y(\omega_2+\omega_1,z_0)}{|k_z(\omega_1)|+|k_z(\omega_2)|+|k_z(\omega_1+\omega_2)|}$$
$$\Big\{-|k_s(\omega_1)|\chi_{yyks}^{(2),D1}(\omega_2+\omega_1,\omega_2,\omega_1,z_0) - |k_s(\omega_2)|\chi_{yyks}^{(2),D2}(\omega_2+\omega_1,\omega_2,\omega_1,z_0)$$
$$+(|k_s(\omega_1)|+|k_s(\omega_2)|)\,\chi_{yyks}^{(2),Q}(\omega_2+\omega_1,\omega_2,\omega_1,z_0)\Big\} \tag{33}$$

$$\chi_{SSP}^{(2)QB}(-\omega_2,-\omega_2-\omega_1,\omega_1) = \sum_{s}^{x,y,z}\sum_{k}^{x,z}\frac{f_k(\omega_1,z_0)f_y(\omega_2,z_0)f_y(\omega_2+\omega_1,z_0)}{|k_z(\omega_1)|-|k_z(\omega_2+\omega_1)|-|k_z(\omega_2)|}$$
$$\Big\{-|k_s(\omega_1)|\chi_{yyks}^{(2),D1}(\omega_2+\omega_1,\omega_2,\omega_1,z_0) + |k_s(\omega_2+\omega_1)|\chi_{yyks}^{(2),D2}(\omega_2+\omega_1,\omega_2,\omega_1,z_0)$$
$$+(|k_s(\omega_1)|-|k_s(\omega_2+\omega_1)|)\,\chi_{yyks}^{(2),Q}(\omega_2+\omega_1,\omega_2,\omega_1,z_0)\Big\} \tag{34}$$

These two expressions can be simplified by noting the transversality of fields, which means the wavevector and field must locally be orthogonal, as described by Eq. 35.

$$\sum_{i}^{x,y,z} k_i(\omega)f_i(\omega)L_{ii}(\omega)E_i(\omega) = \boldsymbol{k}(\omega)\cdot\boldsymbol{E}_{loc}(\omega) = 0 \tag{35}$$

Based on the defined laboratory coordinates, the k-vectors are fixed within the xz-plane. Therefore, the summation over 's' is restricted to x and z, with the y-contributions vanishing.

Furthermore, based on an isotropic symmetry argument, the only non-vanishing susceptibility terms are the YYXX and YYZZ, meaning $s = k$. Based on this, the D1 quadrupolar contribution must vanish along with the $k_s(\omega_1)$ contribution to the Q term. This means the SFG and DFG' expressions can be simplified to Eqs. 36 and 37.

$$\chi_{SSP}^{(2)QB}(\omega_2+\omega_1,\omega_2,\omega_1) = \sum_k^{x,z} \frac{f_k(\omega_1,z_0)f_y(\omega_2,z_0)f_y(\omega_2+\omega_1,z_0)}{|k_z(\omega_1)|+|k_z(\omega_2)|+|k_z(\omega_1+\omega_2)|}$$
$$|k_k(\omega_2)|\left\{-\chi_{yykk}^{(2),D2}(\omega_2+\omega_1,\omega_2,\omega_1,z_0)+\chi_{yykk}^{(2),Q}(\omega_2+\omega_1,\omega_2,\omega_1,z_0)\right\} \quad (36)$$

$$\chi_{SSP}^{(2)QB}(-\omega_2,-\omega_2-\omega_1,\omega_1) = \sum_k^{x,z} \frac{f_k(\omega_1,z_0)f_y(\omega_2,z_0)f_y(\omega_2+\omega_1,z_0)}{|k_z(\omega_1)|-|k_z(\omega_2+\omega_1)|-|k_z(\omega_2)|}$$
$$|k_k(\omega_2+\omega_1)|\left\{\chi_{yykk}^{(2),D2}(\omega_2+\omega_1,\omega_2,\omega_1,z_0)-\chi_{yykk}^{(2),Q}(\omega_2+\omega_1,\omega_2,\omega_1,z_0)\right\} \quad (37)$$

Clearly, there is significant similarity between the two expressions, but they are modulated by different combinations of wavevectors. Specifically, the ratio of the DFG' and SFG responses is given in Eq. 38. It is worth noting that the k-subscripts of the k-vectors on the right side of Eq. 38 have been neglected since both contributions arise from the upconversion beam in each experiment and are thus assumed to have the same incidence angle and minimal dispersion.

$$\frac{\chi_{SSP}^{(2)QB}(-\omega_2,-\omega_2-\omega_1,\omega_1)}{\chi_{SSP}^{(2)QB}(\omega_2+\omega_1,\omega_2,\omega_1)} = \frac{|k_z(\omega_1+\omega_2)|+|k_z(\omega_2)|+|k_z(\omega_1)|}{|k_z(\omega_2+\omega_1)|+|k_z(\omega_2)|-|k_z(\omega_1)|}\frac{|k(\omega_2+\omega_1)|}{|k(\omega_2)|} \quad (38)$$

If one ignores dispersion completely, also taking a collinear beam geometry as in used in the work presented here, then Eq. 38 becomes Eq. 39.

$$\frac{\chi_{SSP}^{(2)QB}(-\omega_2,-\omega_2-\omega_1,\omega_1)}{\chi_{SSP}^{(2)QB}(\omega_2+\omega_1,\omega_2,\omega_1)} \approx \left|\frac{k(\omega_2+\omega_1)}{k(\omega_2)}\right|^2 \quad (39)$$

By considering the DFG response simultaneously generated with the SFG, a similar process yields Eq. 40.

$$\chi_{SSP}^{(2)QB}(-\omega_2+\omega_1,-\omega_2,\omega_1) = \sum_k^{x,z} \frac{f_k(\omega_1,z_0)f_y(\omega_2,z_0)f_y(\omega_2-\omega_1,z_0)}{|k_z(\omega_2-\omega_1)|+|k_z(\omega_2)|-|k_z(\omega_1)|}$$
$$|k_k(\omega_2)|\left\{-\chi_{yykk}^{(2),D2}(\omega_2,\omega_2-\omega_1,\omega_1,z_0)+\chi_{yykk}^{(2),Q}(\omega_2,\omega_2-\omega_1,\omega_1,z_0)\right\} \quad (40)$$

Therefore, by comparison to Eq. 36, the ratio between DFG and SFG is given by Eq. 41, assuming no dispersion. This is purely the ratio of their coherence lengths.

$$\frac{\chi_{SSP}^{(2)QB}(-\omega_2+\omega_1,-\omega_2,\omega_1)}{\chi_{SSP}^{(2)QB}(\omega_2+\omega_1,\omega_2,\omega_1)} \approx \frac{|k_z(\omega_1+\omega_2)|+|k_z(\omega_2)|+|k_z(\omega_1)|}{|k_z(\omega_2-\omega_1)|+|k_z(\omega_2)|-|k_z(\omega_1)|}$$
$$\approx \left|\frac{k(\omega_2+\omega_1)}{k(\omega_2-\omega_1)}\right| \quad (41)$$

One can then simply compare the two DFG responses (DFG and DFG'), as in Eq. 42.

$$\frac{\chi_{SSP}^{(2)QB}(-\omega_2,-\omega_2-\omega_1,\omega_1)}{\chi_{SSP}^{(2)QB}(-\omega_2+\omega_1,-\omega_2,\omega_1)} \approx \left|\frac{k(\omega_2+\omega_1)}{k(\omega_2)}\right|^2 \left|\frac{k(\omega_2-\omega_1)}{k(\omega_2+\omega_1)}\right|$$

$$= \left|\frac{k(\omega_2 + \omega_1)k(\omega_2 - \omega_1)}{k(\omega_2)^2}\right|$$
$$\approx 1 - \frac{k(\omega_1)^2}{k(\omega_2)^2}$$
$$\approx 1 \quad (42)$$

Therefore, the bulk quadrupolar response should show almost no dependence on the upconversion frequency, but does show a significant amplitude scaling factor difference between SFG and DFG pathways.

Experimental SFG and DFG' Comparison

As discussed above, since the SFG and DFG' pathways include the same three frequencies, the intrinsic dipolar contributions are free from dispersion and must be equal. Therefore, any difference between the responses from these two pathways can only arise from depth contributions or signals beyond the electric dipole approximation. Figure S3 shows the experimental spectra for these two pathways for both D_2O and H_2O where a distinct offset in the real part can be seen, just as for the comparison between SFG and DFG shown in Figure 3 in the main text. As this difference is only present in the real part, the two responses clearly have very similar phases, but distinctly different amplitudes. Therefore, since amplitude differences arising from dipolar depth signals are necessarily accompanied by significant phase differences (as discussed above), the origin of this effect must be from signals generated beyond the electric dipolar approximation.

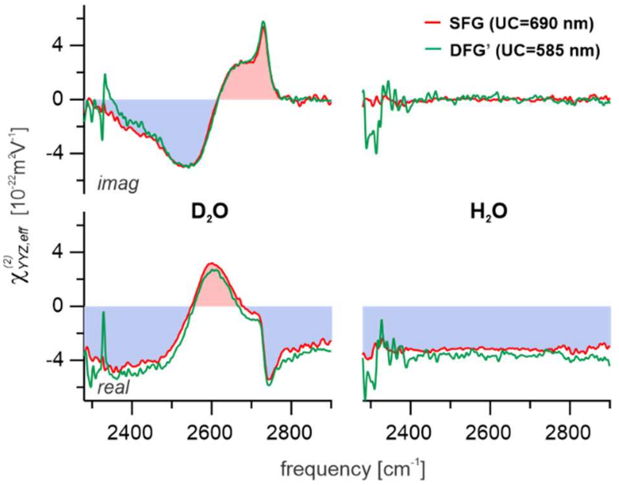

Figure S3: SFG and DFG' spectra for both D_2O and H_2O.

Based on the theoretical comparison between SFG and DFG' for the different quadrupolar contributions above, this difference likely sources from the IQ or QB contributions. Specifically, as shown in Eqs. 27 and 28, the IQ contribution is, in principle, different between SFG and DFG' based on dispersion in the gradient of the dielectric function across the interface. If this dispersion is significant, however, the comparison between DFG and DFG' shown in Figure 4 in the main text would also be expected to show similar dispersion effects. Therefore, as the DFG and DFG' responses are almost exactly equal, the effect of dielectric dispersion can be neglected and the IQ mechanism discounted as the origin of the observed discrepancy. By contrast, the QB contribution inherently gives a significant amplitude difference between SFG and DFG, disregard of dispersion effects. It is this contribution that is hence concluded to be the source of the observed discrepancy.

Analysis of the Measured Water Response

The measured spectra of the non-resonant response from H_2O for both SFG and DFG with an upconversion of 690 nm (i.e., the simultaneous responses) are shown in Figure S4 along with that from the permutation-symmetric DFG' response with an upconversion at 585 nm (i.e., the output SFG frequency). Both real and imaginary parts have been fitted with a constant over the 2450-2750 cm^{-1} frequency range, with the resulting magnitudes of the three responses given in Table S1.

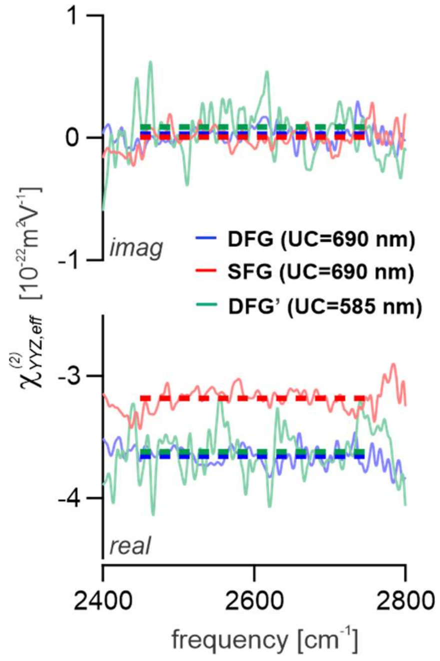

Figure S4: Real and imaginary parts of the non-resonant H_2O spectra for the SFG, DFG, and DFG' (shifted upconversion) pathways. Also shown are the fitted values for this contribution within the 2450-2750 cm^{-1} frequency range.

Table S1: Extracted values for the SFG, DFG, and DFG' susceptibilities from the non-resonant contribution of H_2O.

Susceptibility (upconversion / nm)	Value / $10^{-22}m^2V^{-1}$
$\lvert\chi_{eff}^{SFG}(690)\rvert$	3.1842 ± 0.0046
$\lvert\chi_{eff}^{DFG}(690)\rvert$	3.6555 ± 0.0048
$\lvert\chi_{eff}^{DFG}(585)\rvert$	3.6215 ± 0.0118

From the data, it is clear that there is a significant measurable difference between the SFG response and either DFG response, but only a minute discrepancy between the two DFG responses. As discussed above, this fits well with the expectation from a bulk quadrupolar contribution to the overall signals.

Using the values for the input frequencies, the theoretical ratio between the bulk quadrupolar contributions to these responses can be calculated from the theory outlined above. For the simultaneously generated responses, this is given in Eq. 43.

$$\frac{\chi_{SSP}^{(2)QB}(-\omega_2+\omega_1,-\omega_2,\omega_1)}{\chi_{SSP}^{(2)QB}(\omega_2+\omega_1,\omega_2,\omega_1)} \approx \left|\frac{k(\omega_2+\omega_1)}{k(\omega_2-\omega_1)}\right| \approx 1.437 \tag{43}$$

Taking the measured values from Table S1, the ratio of the total responses can then be determined, as in Eq. 44, where the remaining (entirely interfacial) contributions to the overall response are labelled as *I*, and are equal between SFG and DFG.

$$\frac{\chi_{eff}^{DFG}(690)}{\chi_{eff}^{SFG}(690)} = \frac{\chi_{DFG}^{(2)QB}(690) + \chi_{DFG}^{(2)I}(690)}{\chi_{SFG}^{(2)QB}(690) + \chi_{SFG}^{(2)I}(690)} = 1.1480 \pm 0.0022 \qquad (44)$$

Combining these two equations then allows us to extract the ratio between the bulk quadrupolar contribution and the remaining interfacial contribution, as in Eqs. 45 and 46.

$$1.437\chi_{SFG}^{(2)QB}(690) + \chi_{SFG}^{(2)I}(690) = (1.1480 \pm 0.0022)\left(\chi_{SFG}^{(2)QB}(690) + \chi_{SFG}^{(2)I}(690)\right) \qquad (45)$$

$$\frac{\chi_{SFG}^{(2)QB}(690)}{\chi_{SFG}^{(2)I}(690)} = 0.512 \pm 0.009 \qquad (46)$$

This value of 0.512 thus suggests that the overall SFG response is ~34% bulk quadrupolar, with the corresponding DFG proportion being ~42%.

Whilst in the theory outlined above, we show that the bulk quadrupolar response is mostly independent on upconversion frequency, there is still a slight deviation of this ratio from unity, as is equally observed in the experimental data in Table S1. This allows us to test the conclusions about the bulk quadrupolar contributions to each response by feeding the extracted ratio in Eq. 46 into the theoretical ratio of the two DFG responses and comparing it to the measured value. This comparison is detailed in Table S2 (also shown in Table 1 in the main text) where there is remarkable similarity between the measured and predicted values, with the deviation being well below the uncertainty.

Table S2: Comparison between measured and predicted values of the ratio of H_2O non-resonant amplitudes for DFG responses measured with upconversion beams at 585 and 690 nm.

Susceptibility Ratio	Measured	Predicted
$\frac{\chi_{eff}^{DFG}(585)}{\chi_{eff}^{DFG}(690)}$	0.991±0.003	0.988±0.010

In addition to quantifying the bulk quadrupolar contribution to the non-resonant response, we can also assess its significance to the resonant response. Unlike the non-resonant contribution which is frequency-independent and thus yields a constant value for the individual SFG and DFG spectra and thus also their amplitude ratio, the ratio for the resonant response can be influenced by the vibrational line-shapes of the different source terms, and thus is not generally independent of frequency. Therefore, rather than assessing the quadrupolar contribution by fitting the spectra with a constant, the amplitude ratio between DFG and SFG (shown in Figure S5) must be assessed in terms of its deviation from unity. The spectrum has been fitted with a constant value between 2500-2700 cm^{-1}, representing the average ratio between the spectra within this region of high signal-to-noise. From the fitting, the average comes to 1.00, showing an overall lack of any significant deviation from unity, and thus indicating no significant bulk quadrupolar contributions to the resonant response of the OD stretches of water. Nevertheless, an upper bound can be placed on the significance of any quadrupolar contributions by means of the spread of amplitude ratios within this region. The overall average is then 1.00 ± 0.04, which, based on the predicted amplitude ratio for the bulk quadrupolar contributions of 1.437

shown in Eq. 43 above, shows that their significance to the resonant response must be less than 10%.

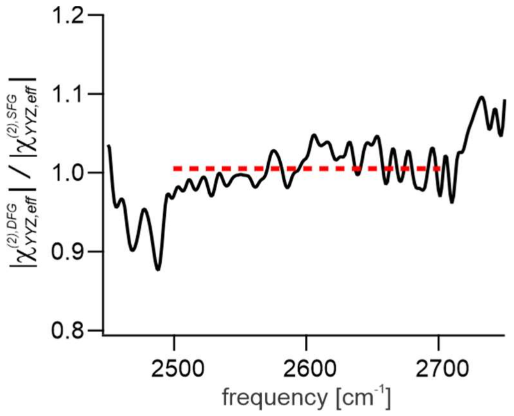

Figure S5: Amplitude ratio (DFG / SFG) of the purely resonant D_2O response, fitted with a constant value.

IQ and IQB Contributions to Resonant and Non-resonant Responses

In contrast to the non-resonant contribution, it is clear that no amplitude difference between SFG and DFG is observed in the resonant response, and thus any bulk quadrupolar contribution must be negligible. However, as the ID, IQ, and IQB contributions to SFG and DFG are equal, a comparison between their spectra cannot separate their relative contributions. Nevertheless, with the QB term only being significant in the non-resonant response, the quadrupolar mechanisms are suggested to be dominated by interactions with the electronic part of the molecular wavefunction rather than its vibrational component. Therefore, as shown in the theoretical description above, the IQ and IQB mechanisms are also suggested to be insignificant contributions to the resonant response as they arise from the same intrinsic mechanisms i.e., D1, D2, and Q. This suggests that the resonant response is predominantly dipolar in origin and thus reports on the structural anisotropy at the interface.

For the non-resonant response, however, it is not immediately clear how much of the response arises from the ID, IQ, and IQB mechanisms, with them potentially all being significant contributors alongside the QB term. As discussed in the main text, however, the discrepancy between the observed anisotropic depths for the resonant and non-resonant responses (7.7 vs 3.1 Å, respectively) does suggest that they arise from different mechanisms. Whilst this, to some extent, is expected as they probe different parts of the molecular wavefunction: vibrational vs. electronic, with the observation that the anisotropy seems to be predominantly due to the change in H-bonding environment, it is likely that the anisotropy in molecular vibrational structure and the electron cloud are similar. Therefore, the reduced depth of the non-resonant response may well instead be an indicator for interfacial quadrupolar contributions since these are dominated by the anisotropy in the dielectric function at the interface. This conclusion also fits well with previous suggestions by Tahara and co-workers who, through polarisation-dependent measurements, determined that the non-resonant contributions from air-liquid interfaces includes a substantial IQ contribution.(*5*) We speculate, therefore, that the non-resonant response of water is predominantly quadrupolar, with an interfacial contribution that only depends on the input frequencies through their dielectric functions and a bulk contribution that is almost frequency-independent, but depends on incident angle owing to the z-component of the wave-vector mismatch and the specific pathway being probes (SFG or DFG).

The Non-resonant Response as a Phase Reference

As discussed in the main text, the non-resonant response from water has been suggested as a possible phase reference for SFG. Despite its exact phase being previously contentious, it is shown here to be very close to ±180°. Specifically, with an apparent depth of 3.1Å, the overall SFG response has a phase of -179.6°. To make a good phase reference, its phase should be well-known and largely independent of the specific experimental set-up, including the incident angles and input frequencies. While a non-zero depth means the response is dependent on the coherence length and thus on both of the above experimental factors, with anisotropic contributions that decay over well below 1 nm, such dependency is negligible. Furthermore, the bulk quadrupolar response is precisely always 180° in phase. Therefore, the combination of small-depth interfacial terms with the BQ response makes the non-resonant contribution an excellent phase reference. By contrast, the amplitude of the non-resonant response will be both frequency and incident angle dependent owing to the frequency-dependence of the dielectric function and wavevector mismatch, as well as the obvious dependence of the incidence angle on the z-projection of the latter.

Frequency Dependence of the SFG-DFG Phase Difference

Figure 5(a) in the main text shows the extracted phase difference between the SFG and DFG spectra for both the non-resonant response of H_2O and the purely resonant response of D_2O. When assessing the resulting spectra, both responses are fitted with constant values to obtain their average and extract their corresponding anisotropic decay lengths. Whilst this approach is entirely representative of the non-resonant response as this should be independent of frequency, it neglects any possible frequency-dependence of the phase difference for the resonant response. This, for example, could arise from differing decay lengths for the different vibrational resonances corresponding to specific structural motifs. On closer inspection, the resonant phase difference reveals interesting features which could indeed be indicative of the different structural motifs having differing spatial distributions. Specifically, it appears that the free OD species which gives rise to the resonance at 2740 cm^{-1} has a phase difference of ~0º, suggesting it is only present in the top-most layer, in accordance with expectation. In contrast, the hydrogen bonded mode at ~2540 cm^{-1} reports a phase difference of ~1º, suggesting it originates from slightly below the phase boundary. However, when comparing the phase differences for the resonant and non-resonant responses, they both show similar amplitude deviations from their respective mean values. This suggests that the extracted phase difference spectra may contain appreciable distortions (systematic errors). Therefore, for more conclusive insight into such frequency dependence, more experimental work and analysis would be required at even higher precision, which is currently beyond our experimental capabilities.

References

1. A. P. Fellows, V. Balos, B. John, Á. Díaz Duque, M. Wolf, M. Thämer, Obtaining extended insight into molecular systems by probing multiple pathways in second-order nonlinear spectroscopy. *J. Chem. Phys.* **159**, 164201 (2023).

2. V. Balos, T. Garling, A. D. Duque, B. John, M. Wolf, M. Thämer, Phase-Sensitive Vibrational Sum and Difference Frequency-Generation Spectroscopy Enabling Nanometer-Depth Profiling at Interfaces. *J. Phys. Chem. C* **126**, 10818–10832 (2022).

3. M. Thämer, T. Garling, R. K. Campen, M. Wolf, Quantitative determination of the nonlinear bulk and surface response from alpha-quartz using phase sensitive SFG

spectroscopy. *J. Chem. Phys.* **151**, 64707 (2019).

4. A. Morita, *Theory of Sum Frequency Generation Spectroscopy* (Springer, Singapore, 2018).

5. S. Yamaguchi, K. Shiratori, A. Morita, T. Tahara, Electric quadrupole contribution to the nonresonant background of sum frequency generation at air/liquid interfaces. *J. Chem. Phys.* **134**, 184705 (2011).
